# Design and Engineering of an Artificial Bifunctional *N*‐Deacetylase/*N*‐Sulfotransferase for the Biosynthesis of *N*‐Sulfated Heparosan

**DOI:** 10.1002/advs.76499

**Published:** 2026-07-10

**Authors:** Xintong Xi, Ruirui Xu, Daoan Wang, Guobin Yin, Xinjing Wang, Haiqin Chen, Guocheng Du, Jian Chen, Zhen Kang

**Affiliations:** ^1^ The Key Laboratory of Carbohydrate Chemistry and Biotechnology Ministry of Education Jiangnan University Wuxi China; ^2^ School of Food Science and Technology Jiangnan University Wuxi China; ^3^ The Science Center for Future Foods Jiangnan University Wuxi China; ^4^ Jiangsu Province Basic Research Center For Synthetic Biology Jiangnan University Wuxi China

**Keywords:** artificial bifunctional enzyme, Escherichia coli, heparin, N‐deacetylase/N‐sulfotransferase, N‐sulfated heparosan, polysaccharide

## Abstract

Heparin is a widely used anticoagulant, while its traditional animal‐derived production faces significant challenges in safety and scalability. The active expression of the initiating‐step bifunctional enzyme *N*‐deacetylase/*N*‐sulfotransferase (NDST) in bacteria remains a key bottleneck in heparin biosynthesis. Here, we firstly mined and characterized diverse polysaccharide *N*‐deacetylases (NDases) in *Escherichia coli* and revealed several NDases that are active toward the deacetylation of heparosan. An artificial ND*^Dr^*‐RL‐ST was designed and constructed by fusing an active NDase with a heterogenous NST domain. The ND*^Dr^* ^–MEc^/ST variant with a 7.48‐fold improvement in catalytic efficiency was engineered by combining dynamic cross‐correlation analysis, sequence consensus analysis, and mutation‐effect prediction with sequential linker optimization and rational mutagenesis, resulting in enhanced substrate binding, active‐site stabilization, and improved inter‐domain coordination. ND*^Dr^* ^–MEc^/ST efficiently converted heparosan to *N*‐sulfated polysaccharide with a sulfation degree of 82.8%. The results highlighted the critical role of *N*‐sulfotransferase interaction with ND*^Dr^* domain in stabilizing the catalytic conformation essential for bifunctional enzyme activity. Moreover, this work also provided valuable insights for the rational design of artificial multifunctional enzymes for polysaccharide modification.

## Graphical abstract

An artificial bifunctional *N*‐deacetylase/*N*‐sulfotransferase was engineered in *Escherichia coli* by combining screened *N*‐deacetylases with an NST domain. The integrated engineering strategy improved enzyme performance. The optimized enzyme efficiently converted heparosan into *N*‐sulfated heparosan, addressing a key bottleneck in microbial heparin biosynthesis and enabling scalable polysaccharide modification.

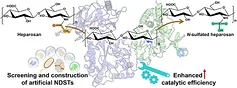

## 1 Introduction

Heparin is a naturally occurring, highly sulfated glycosaminoglycan that represents the most extensively used anticoagulant in clinical medicine. Beyond its anticoagulant function, heparin exhibits diverse pharmacological activities, including antithrombotic, antitumor, anti‐inflammatory, and antiviral effects [1, 2, 3, 4]. Traditionally, heparin is extracted from natural animal tissues, especially the porcine intestinal mucosa. However, this approach faces significant challenges, including the risk of contamination with heterogeneous glycosaminoglycans during the extraction process, as well as the inherent vulnerability of the supply chain [5, 6]. Therefore, bioengineering‐based production of heparin offers a promising alternative to overcome the limitations associated with animal‐derived heparin [7, 8].

In biosynthetic systems, heparosan—a polysaccharide composed of repeating ‐GlcA‐GlcNAc‐ disaccharide units—serves as the initial substrate, undergoing a series of enzymatic modifications catalyzed sequentially by *N*‐deacetylase/*N*‐sulfotransferase (NDST), C5‐epimerase, 2‐*O*‐sulfotransferase, 6‐*O*‐sulfotransferase, and 3‐*O*‐sulfotransferase with the sulfonate group donor 3′‐phosphoadenosine‐5′‐phosphosulfate (PAPS), ultimately producing heparin, which is characterized by its highly sulfated and structurally complex architecture [9, 10, 11]. The anticoagulant activity of heparin primarily depends on a specific core pentasaccharide motif, GlcNS/Ac6S (1→4) GlcA (1→4) GlcNS3S6S (1→4) IdoA2S (1→4) GlcNS6S, which exhibits high‐affinity binding to antithrombin III [12]. Among these modifications, the generation of *N‐*sulfoglucosamine (GlcNS) units by NDST—through coupling *N‐*deacetylation and *N*‐sulfation of glucosamine residues—represents the critical initiating step of the cascade [13, 14].

Although chemoenzymatic synthesis has been explored as an alternative route, where heparosan is first treated with NaOH for *N‐*deacetylation, followed by enzymatic treatment for *N‐*sulfation [15, 16]. However, NaOH treatment not only completely eliminates the acetyl groups in heparosan but also substantially impairs its structural architecture [17]. In contrast, the bifunctional enzyme NDST, which integrates both *N*‐deacetylase (NDase) and *N*‐sulfotransferase (NST) activities, plays a pivotal role as the initiating and rate‐limiting catalyst in heparin biosynthesis, since its dual functions are indispensable for generating GlcNS units that enable subsequent modification steps [18]. To date, four human NDST isoforms have been identified, and the structural and mechanistic features of NDST1 have been elucidated in detail [19, 20]. In a notable strategy, Deng et al. introduced the unnatural monosaccharide *N‐*trifluoroacetylglucosamine into *Escherichia coli* to bypass the *N*‐deacetylation step, thereby facilitating downstream sulfation modifications, effectively circumventing the natural function of NDase [21]. In our previous work, we successfully separated the bifunctional NDST into its individual domains, achieving high‐level expression of the NST domain in *E. coli* [22]. During the preparation of this manuscript, Li et al. concurrently engineered a prokaryote‐compatible NDST variant in *E. coli* [23]. Collectively, these advances highlight the growing efforts toward bacterial expression of NDST.

In this study, we aimed to engineer an artificial NDST with efficient heterologous expression in *E. coli*. To this end, multiple NDase domains were expressed and systematically characterized to evaluate their compatibility with the NST domain and to achieve functional integration. Building on the resulting bifunctional enzyme NDST, we developed a rational, integrated strategy combining dynamic cross‐correlation analysis, sequence consensus analysis, and mutation‐effect prediction to accelerate the development of high‐activity enzymes. Finally, the engineered variant ND*^Dr^* ^–MEc^/ST with high activity was applied to the enzymatic production of *N*‐sulfated heparosan.

## 2 Results

### 2.1 Screening and Active Expression of *N*‐Deacetylase in *E. coli* for Heparosan Deacetylation

*N*‐deacetylation represents the initial step in heparin structural modification, where NDase catalyzes the removal of *N*‐acetyl groups from *N*‐acetylglucosamine (GlcNAc) residues of heparosan to enable subsequent sulfation. Yet, native heparosan‐specific NDases have not been functionally expressed in prokaryotic hosts, posing a major bottleneck for both structural modification and de novo biosynthesis of heparin. Therefore, re‐evaluating and screening NDases that are amenable to expression in prokaryotic systems is of considerable importance. Moreover, several deacetylases expressed in prokaryotic hosts have been reported to act on other GlcNAc‐containing polysaccharides (e.g., chitin) as well as other acetylated polysaccharides (e.g., acetyl xylan and peptidoglycan) [24, 25, 26, 27, 28]. This led us to hypothesize that such NDases may also serve as potential candidates for catalyzing the deacetylation of heparosan. To this end, we first selected three heparosan‐specific NDases, including ND*^Pv^*, ND*^Dr^*, and ND*^Hs^*. To broaden the candidate pool, we analyzed enzymes targeting acetylated polysaccharides from the UniProt and CAZy databases to identify those with potential heparosan‐deacetylating activity (Figure 1A,B). This search yielded four enzymes from carbohydrate esterase family 4 (CE4 family), ND*^Sp^*, ND*^Mr^*, ND*^Ar^*, and ND*^Sl^*, as well as ND*^Af^*, a CE18 family member reported to share structural similarity with heparosan‐specific NDases [29]. Phylogenetic analysis revealed that the heparosan‐specific NDases clustered more closely with CE18 ND*^Af^* than with CE4 NDases (Figure 1C).

**FIGURE 1:**
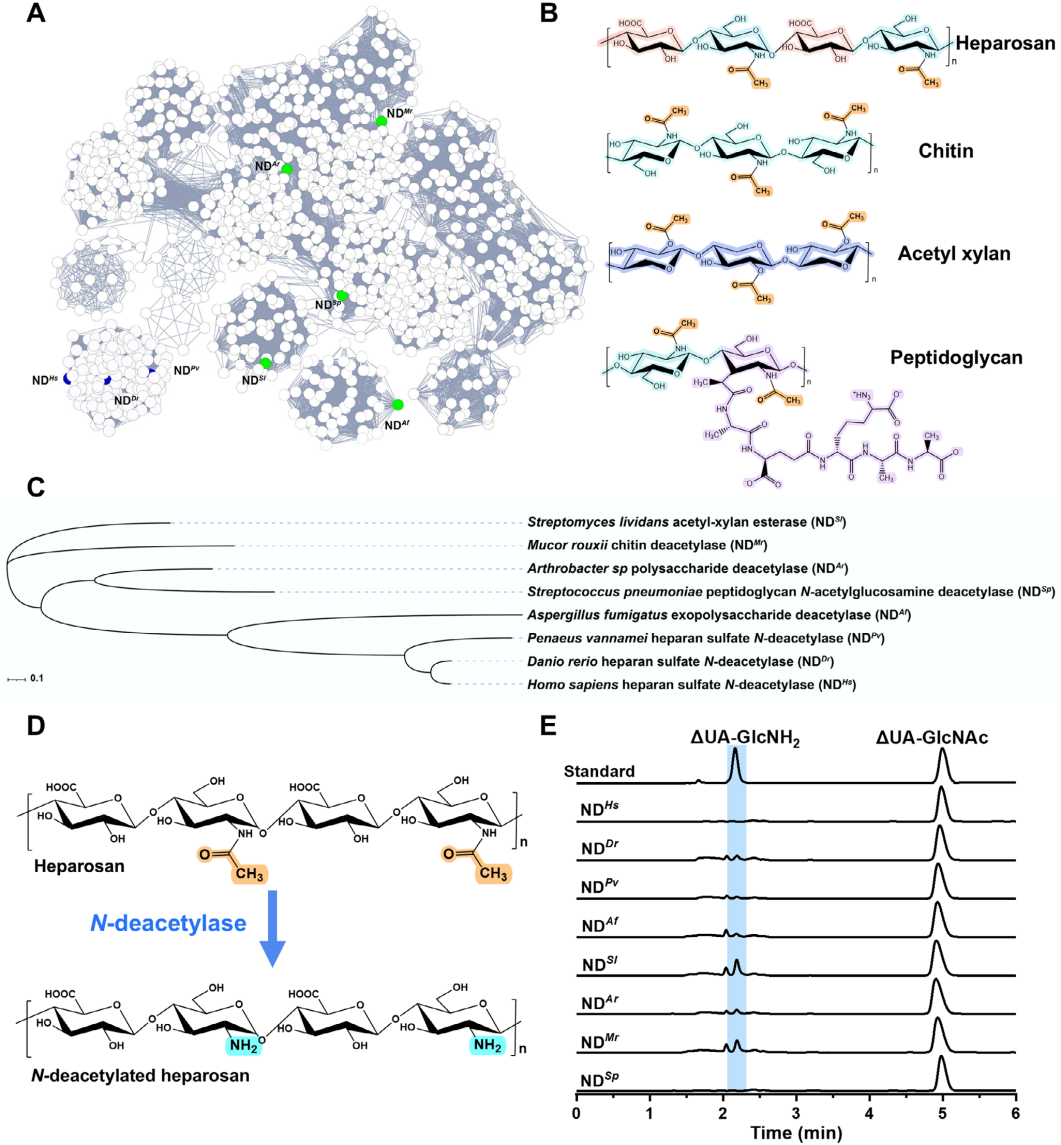
Screening and characterization of *N‐*deacetylases targeting heparosan. (A) Bioinformatic analysis of NDases genes from the UniProt and CAZy databases through Cytoscape. (B) Natural polysaccharide substrates of the selected NDases. (C) Phylogenetic analysis of the selected sequences. (D) Schematic representation of the *N*‐deacetylation reaction. (E) HPLC analysis of *N*‐deacetylated heparosan disaccharides catalyzed by NDases.

To evaluate their catalytic potential, the eukaryote‐derived NDase genes were heterologously expressed in *E. coli* Rosetta (DE3) using the expression vector pET32a with solubility‐enhancing tag MBP, followed by assessment of the *N*‐deacetylation activity of the recombinant enzymes toward heparosan (Figure 1D). Products were characterized by high‐performance liquid chromatography (HPLC), revealing that, except for ND*^Sp^* and ND*^Hs^*, *N*‐deacetylated products were detected for all other NDases. These products eluted at approximately 2 min, matching the retention time of the disaccharide standard (Figure 1E). Remarkably, ND*^Mr^* and ND*^Sl^* generated more intense product peaks than NDases naturally acting on heparosan (ND*^Dr^* and ND*^Pv^*), suggesting superior catalytic efficiency and greater potential for *N*‐sulfated heparosan biosynthesis. The catalytic capability of the selected NDases was further evaluated using chitin oligosaccharides as alternative substrates. Several CE family NDases exhibited detectable activities toward triacetylchitotriose or pentaacetylchitopentaose, whereas no detectable deacetylated product was observed for heparosan‐specific NDases ND*^Hs^*, ND*^Dr^*, and ND*^Pv^* (Figure S1), suggesting broader substrate promiscuity among the CE family NDases. Collectively, these findings substantially expand the repertoire of NDases amenable to prokaryotic expression, thereby broadening the toolkit available for heparosan modification.

### 2.2 Assembly and Adaptation of Artificial *N*‐Deacetylase/*N*‐Sulfotransferases

To verify whether the selected NDases could effectively deacetylate heparosan and to further characterize their catalytic activity (the principle of enzyme activity assay is shown in Figure S2), we employed a cascade reaction in which the *N*‐deacetylated products were directly subjected to *N*‐sulfation using an engineered NST from our previous work (Figure 2A). As anticipated, ND*^Mr^* displayed the highest activity among the tested enzymes, followed in order by ND*^Sl^*, ND*^Ar^*, ND*^Dr^* and ND*^Pv^* (Figure 2B). Consistent with these results, HPLC analysis showed *N*‐sulfated disaccharide peaks at around 8 min (Figure 2C), which corresponded well with the trends observed in Figures 1E and 2B. These findings confirmed that the selected NDases are capable of producing *N*‐sulfated heparosan through cascade catalysis with NST. Although the above results were encouraging, the catalytic processes exhibited relatively low enzymatic activity even after systematic optimization of reaction conditions (Figure S3). In nature, heparin *N*‐deacetylases and *N*‐sulfotransferases are often fused into bifunctional enzymes, enabling the rapid completion of the two‐step reaction. Inspired by this, we sought to construct artificial NDSTs by mimicking the natural architecture, employing the previously engineered NST as the C‐terminal domain and linking the *N*‐deacetylases to it via a **r** igid EAAAK **l** inker (abbreviated as RL) (Figure 2D and Figure S4).

**FIGURE 2:**
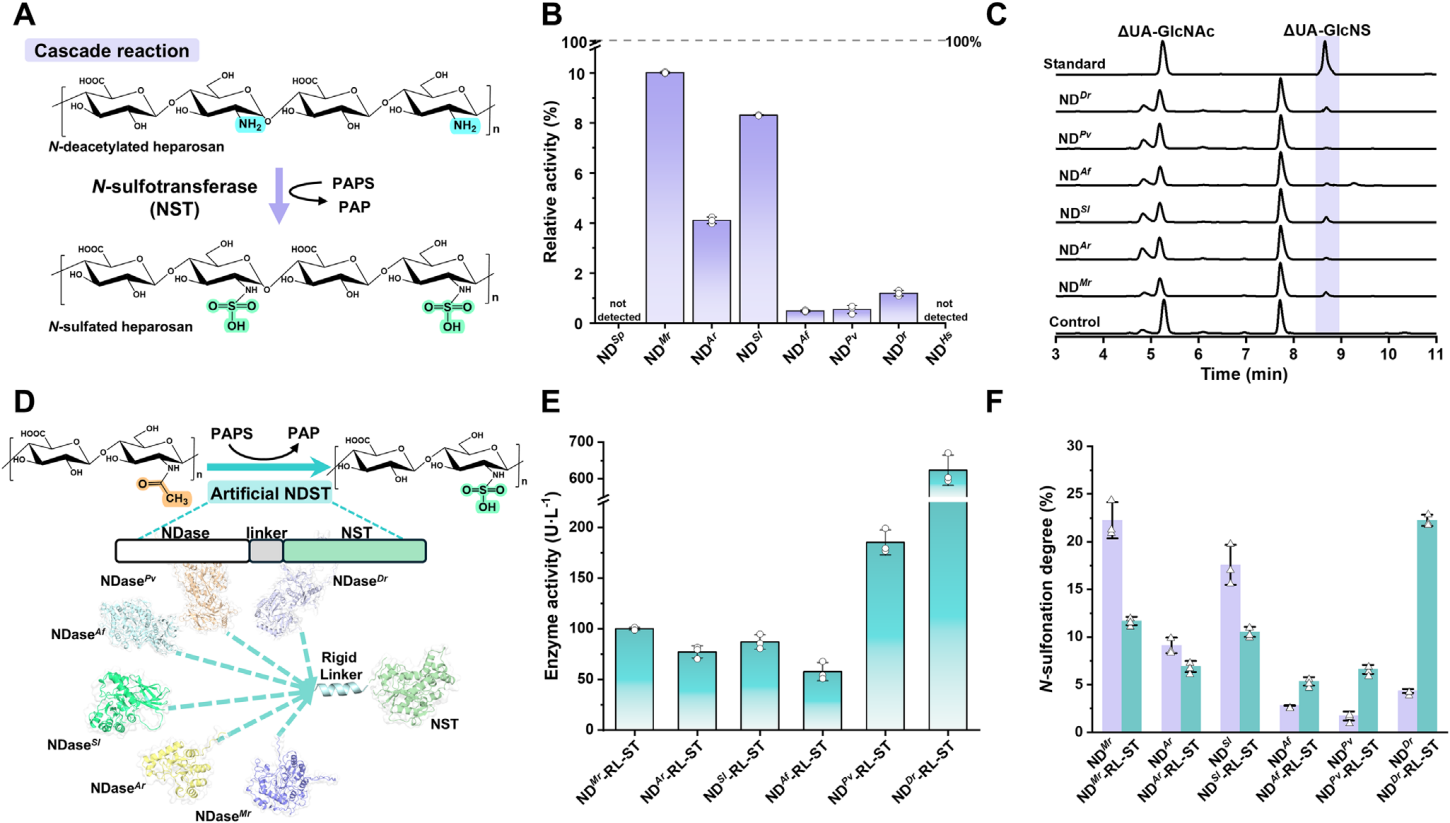
Construction and characterization of the artificial NDST. (A) Schematic representation of cascade *N*‐sulfation using *N*‐deacetylated heparosan. (B) Assessment of NDase activity by coupled *N*‐sulfation reaction. Taking advantage of the colorimetric properties of NST, we quantitatively evaluated the enzymatic activity of NDases by defining the activity of NST toward chemically *N*‐deacetylated heparosan as 100%. (C) HPLC analysis of *N*‐sulfated heparosan disaccharides. (D) Schematic of the artificial NDSTs construction using different NDases and NST. (E) Enzyme activity of the artificial NDSTs toward heparosan. (F) *N‐*sulfation comparison of catalyzed products.

Unexpectedly, the enzyme activity assays produced results opposite to those anticipated. While ND*^Mr^* performed best in the cascade reaction, its advantage was lost when incorporating into the artificial bifunctional enzyme ND*^Mr^*‐RL‐ST. On the contrary, ND*^Dr^*‐RL‐ST and ND*^Pv^*‐RL‐ST displayed markedly higher activities, with ND*^Dr^*‐RL‐ST reaching 623.09 U·L^−1^ (Figure 2E). Further analysis of the *N*‐sulfation degree of the products generated by these artificial enzymes revealed a general decline in sulfation efficiency compared to their individual catalytic domains (Figure 2F and Figure S5). For instance, the *N*‐sulfation degree of ND*^Mr^*‐RL‐ST products decreased to 11.7%, with a similar trend observed for ND*^Sl^*‐RL‐ST. Notably, ND*^Dr^*‐RL‐ST showed the best overall performance, achieving an *N*‐sulfation degree of 22.2%, higher than that of ND*^Dr^* in cascade catalysis, as supported by the comparative time‐course analysis of sulfation degree (Figure S6).

### 2.3 Fusion With the NST Domain Stabilizes the Open Conformation of the Catalytic Pocket of ND*^Dr^*

To elucidate why NDases exhibit divergent catalytic trends when incorporating into artificial bifunctional enzymes compared with their standalone forms, we directly compared the performance of the artificial NDSTs (without the presence of PAPS) with their corresponding NDase domains alone on deacetylation of heparosan hexasaccharide (HN6^AN^) (Figure 3A). Interestingly, no catalytic products were detected for ND*^Dr^* acting on HN6^AN^ in the single‐enzyme form. In contrast, ND*^Dr^*‐RL‐ST exhibited markedly enhanced catalytic performance, producing substantial amounts of deacetylated HN6^AN^ ([M−H]^−^ at *m/z* 1094.3) (Figure 3B,C). We speculate that the NST domain may assist in binding short‐chain substrates, thereby facilitating ND*^Dr^* catalysis. Although the rigid linker exhibited relative effectiveness, the overall activity of ND*^Mr^*‐RL‐ST remained minimal (Figure S7). Moreover, the ND*^Mr^* activity in the one‐pot reaction—where both ND*^Mr^* and NST were introduced simultaneously into the system—was substantially suppressed compared with that in the cascade reaction (Figure S8), suggesting functional interference by the NST domain.

**FIGURE 3:**
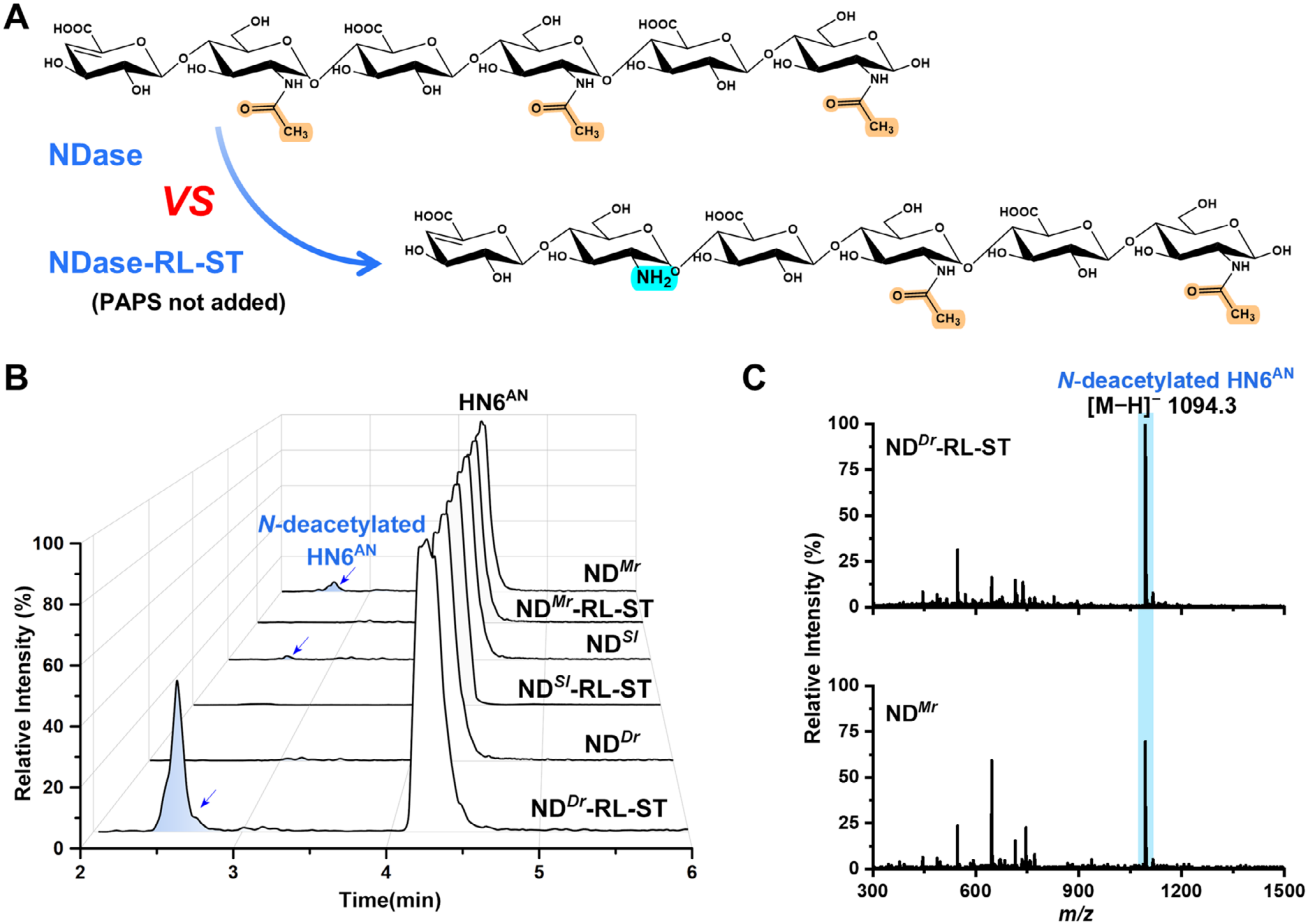
Deacetylation analysis of heparosan oligosaccharides catalyzed by NDases. (A) Schematic representation of oligosaccharide deacetylation. (B) Ultra‐performance liquid chromatography (UPLC) chromatograms and (C) mass spectra of the *N*‐deacetylated HN6^AN^.

Accordingly, the interaction between NDases and NST was further investigated. Obviously, the individual NST domain interacts with the key substrate‐binding residues of ND*^Mr^*, including Tyr255, Asp165 and His218, which may reduce substrate‐binding efficiency (Figure S9A). A similar phenomenon was also observed in ND*^Sl^*, where interactions with Tyr144, Asp168 and Trp198 are critical for substrate binding and catalytic performance (Figure S9B). In contrast, the domains ND*^Dr^* and NST in the artificial enzyme ND*^Dr^*‐RL‐ST displayed a distinct interaction mode. The NST domain interacts with residues Asp583, Pro584, Trp586, Asp588, Glu591, Lys603, Thr604, Cys605 and Asp606 that locates in the loop region of ND*^Dr^* (Figure 4A). This interaction network facilitated the involvement of Arg594 in shaping the ND*^Dr^* active pocket (Figure 4B), which dynamic opening and closing are critical for forming an accessible catalytic pocket. In the single‐enzyme form, the loop may adopt a closed conformation, causing residues such as Arg594 to obstruct the active‐site entrance and reducing catalytic efficiency. In the bifunctional context, the interaction between ND*^Dr^* and NST appeared to stabilize the loop in an open conformation, promoting active‐pocket formation and improving catalytic performance (Figure 4C).

**FIGURE 4:**
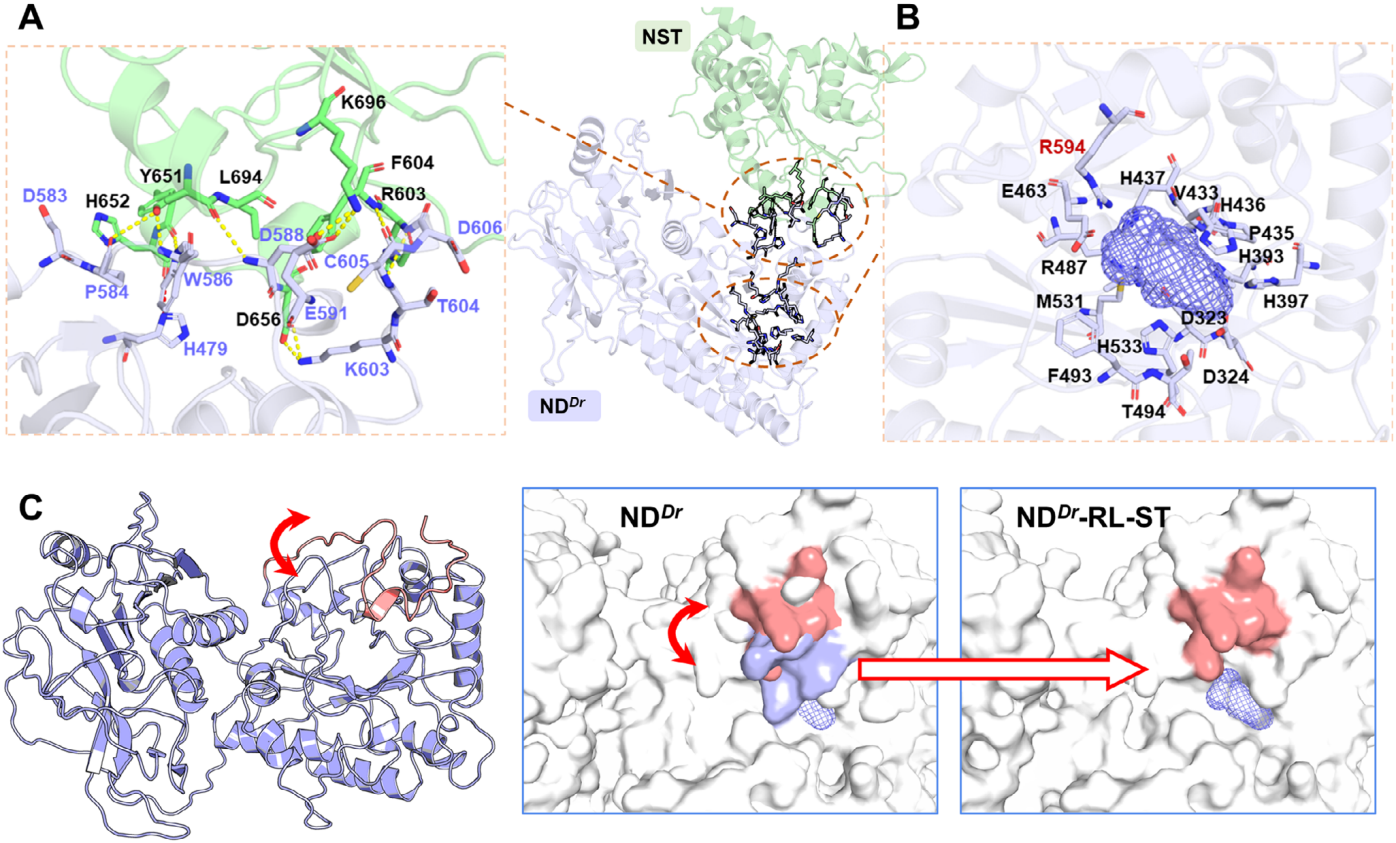
Structural analysis of interactions between the ND*^Dr^* and NST domains. (A) Residue interaction networks formed at ND*^Dr^* and NST domains. The residues from ND*^Dr^* domain are represented by the color light‐blue, and those from NST are represented by the color green. Yellow dashed lines indicate the formed hydrogen bonds. (B) Catalytic pocket formed in the ND*^Dr^* domain. The mesh indicates the formed catalytic pocket. (C) Structural comparison of the loop region in the ND*^Dr^* and ND*^Dr^*‐RL‐ST, highlighting its potential impact on catalytic pocket formation, with the loop region colored salmon.

### 2.4 Optimization of the Linker Region to Improve Enzymatic Activity

In multi‐domain enzymes, linkers are frequently introduced to modulate the spatial distance between domains, with both their length and rigidity strongly affecting catalytic performance [30]. Therefore, we first optimized the linker between MBP and ND*^Dr^* domain (Figure 5A). As shown in Figure 5B, a flexible linker was optimal for maintaining activity. Substitution with rigid linkers (EAAAK)_2_, (PT)_3_, (PT)_4_ reduced activity by 59.1%, 53.6%, and 29.2%, respectively, compared to the flexible control G4S. Replacement with another flexible linker (G6) also led to a 51.7% decrease. Simply extending the G4S linker ((G4S)_2_) slightly reduced activity (−7.2%), while the absence of a linker caused the most severe decline (−76.2%). In addition, we examined the linker connecting ND*^Dr^* and NST domains. The original linker was systematically replaced, both flexible (G4S, G6) and rigid linkers ((PT)_3_, (PT)_4_) were tested. When the flexible (G4S, G6) linkers were used, the increased enzyme activity by 12.7% and 80.9%, respectively, compared to the rigid control (EAAAK) (Figure 5C). However, the highest activity (2132.1 U·L^−1^) was observed in the absence of a linker, corresponding to a 242.2% increase relative to the control enzyme ND*^Dr^*‐RL‐ST. Furthermore, we hypothesized that the C‐terminal sequence of ND*^Dr^* domain (residues 579–664) may function as part of a natural linker regulating interactions between the ND*^Dr^* and NST domains. To test this, a comprehensive UniProt database search was performed, followed by multiple sequence alignment and frequency analysis. For example, position 579 is occupied by Pro in ND*^Dr^*, while Ser appears at high frequency at this site, whereas Asn and Gln are less common (Figure 5D). Based on this analysis, Pro579 was substituted with Ser, Asn, and Gln for comparison. In total, 20 mutants were constructed according to the sequence frequency results. Among them, the R582V variant exhibited remarkably improved performance relative to the parental bifunctional enzyme ND*^Dr^*/ST (hereafter designated as ND*^Dr–^* ^WT^/ST), reaching an activity of 2886.73 U·L^−1^, corresponding to a 35.4% increase in activity.

**FIGURE 5:**
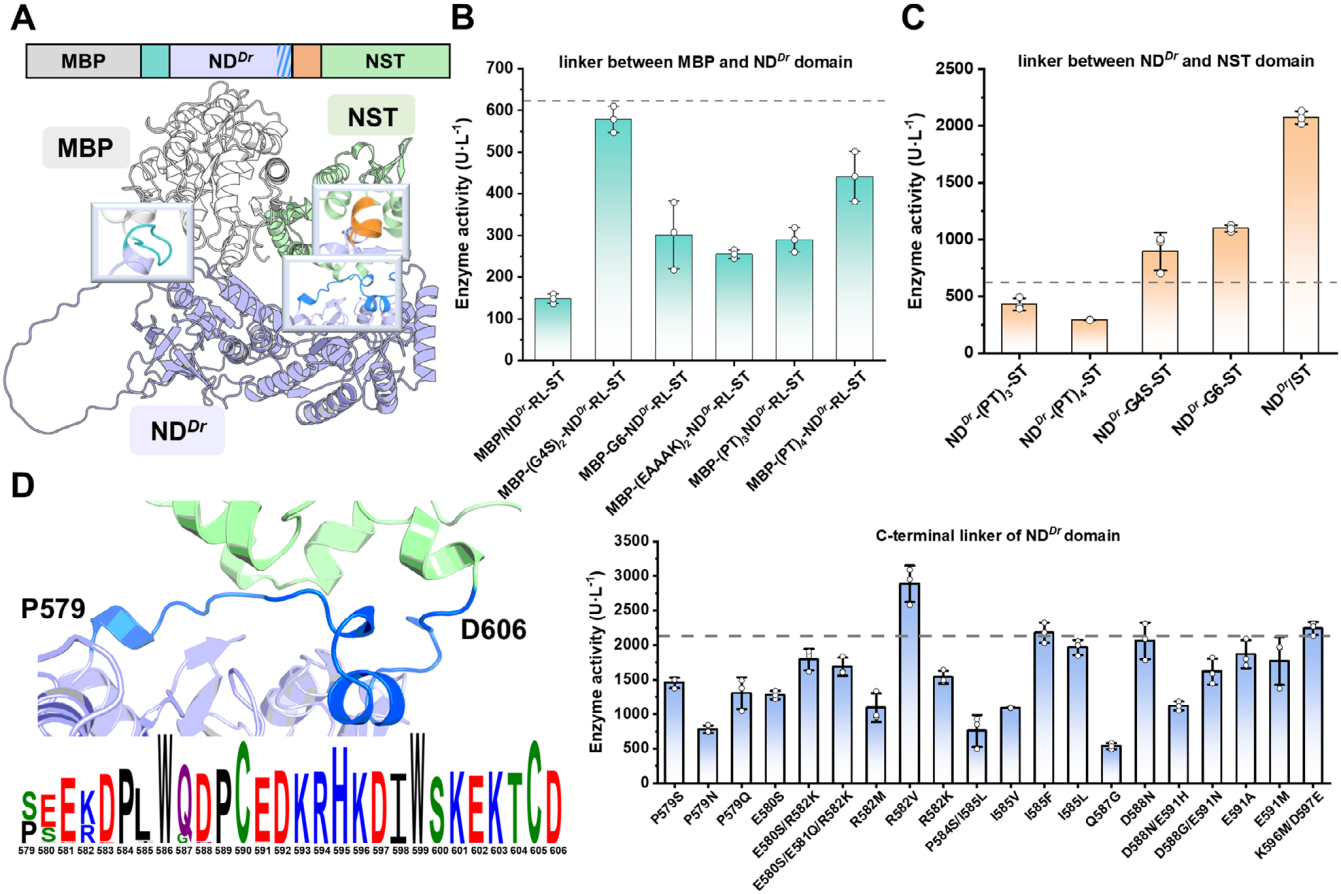
Linker optimization improves artificial bifunctional activity. (A) Schematic representation of sequence optimization across the multiple domains of the bifunctional enzyme. (B) Optimization of the insertion sequence between MBP and ND*^Dr^* domains. (C) Insertion sequence optimization between the ND*^Dr^* and NST domains. (D) Optimization of the C‐terminal sequence in the ND*^Dr^* domain based on sequence analysis.

### 2.5 Dynamics‐ and Sequence‐Guided Mutagenesis for Optimizing the Catalytic Efficiency

A combined mutagenesis strategy, integrating dynamic cross‐correlation matrix (DCCM) analysis based on molecular dynamics (MD) simulations, sequence consensus analysis, and mutation‐effect prediction, was applied to enhance the catalytic efficiency of the bifunctional enzyme (Figure 6A). Firstly, DCCM analysis was performed to assess the residue‐level dynamic correlation across the protein, with particular emphasis on correlations involving catalytic residues to guide the selection of candidate mutation sites (Figure 6B,C). For example, His393, one of the catalytic residues in the ND*^Dr^* domain, exhibited strong positive dynamic correlations with distal residues such as Ser480 and Ser483. Candidate residues for mutagenesis were then identified based on amino acid distribution patterns revealed by sequence consensus analysis (e.g., S480N and S483M). Finally, EV mutation, an unsupervised statistical method, was used to predict the quantitative effects of amino acid substitutions, and mutant with high prediction scores were further select for experimental validation (Figure 6D) [31]. As shown in Figure 6E, a total of 54 variants were generated through site‐directed mutagenesis (D110E, I111V, G118S, Q189L, F219H, F219Y, K222R, A223S, A223P, R224S, R224G, E227L, H228K, H228P, P230V, D234E, T244S, R253K, G254T, G254L, R255K, R255Q, A259S, S260I, S260V, A269T, A269S, L298F, A313P, A313S, A313G, N351T, A369T, Y384H, S385A, S385V, H417K, N428D, N428H, N428R, I443V, F473T, F473Y, V478I, S480N, S483M, G505S, S506P, L512S, L512I, S561R, S561P, S561K, and I577L). Among these, the F219H variant exhibited the greatest enhancement, with an 88.6% increase in activity relative to the control. Variants N428H, S480N, and S506P also exhibited an elevated activity, with increase of 60.2%, 73.8% and 64% compared to the control, respectively.

**FIGURE 6:**
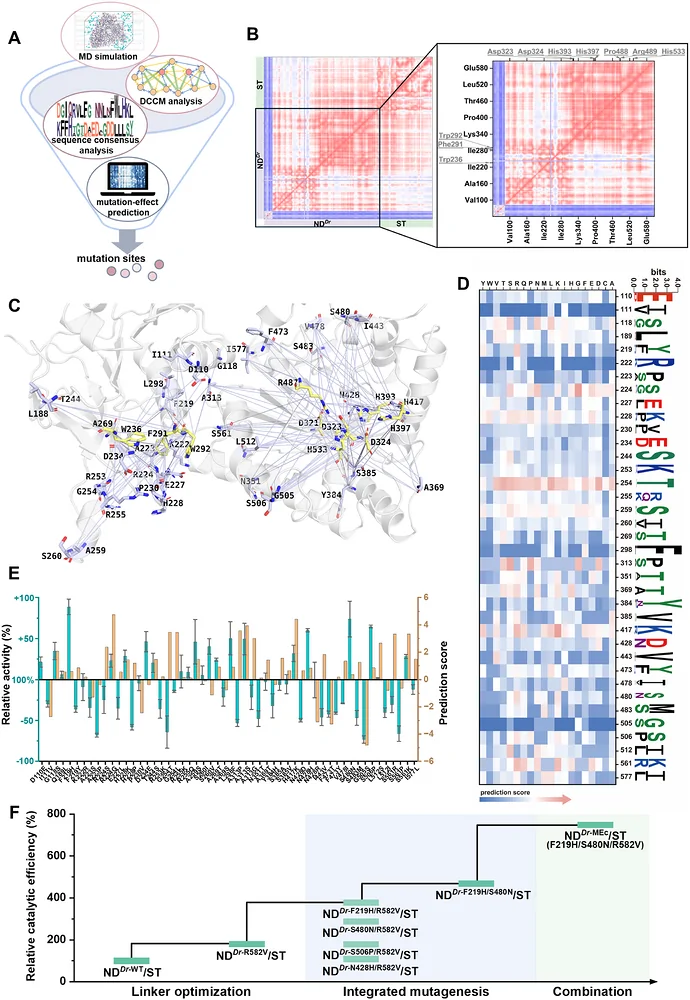
Engineering ND*^Dr^*/ST efficiency through a combined mutagenesis strategy. (A) Workflow of the integrated mutagenesis strategy for enhancing enzyme stability and activity. (B) DCCM analysis of the ND*^Dr^*/ST, with the ND*^Dr^* domain shown in the magnified inset. (C) Schematic representation of mutation sites relative to the catalytic residues in the ND*^Dr^* domain. Catalytic residues in the ND*^Dr^* domain are shown as yellow sticks, while mutation sites are shown as light‐blue sticks. (D) Sequence consensus analysis of candidate mutations and the corresponding mutational landscape predicted by EVmutation. The color scale (blue to red‐brown) indicates increasingly favorable substitutions. (E) Comparison of experimentally determined relative activities (cyan) and prediction scores (orange) for selected mutation sites. Relative activities were normalized to ND*^Dr^* ^–R582V^/ST (100%). (F) Engineering strategy for the stepwise enhancement of the catalytic efficiency of ND*^Dr^*/ST, as evaluated based on kinetic parameters.

Kinetic properties were characterized using purified enzymes (Figure S10), with results summarized in Table 1. The ND*^Dr^* ^–R582V^/ST exhibited a 1.83‐fold increase in catalytic efficiency (*k*_cat_/*K*_m_) relative to the ND*^Dr^* ^–WT^/ST. Mutations at positions 219 and 480 further enhanced performance compared to ND*^Dr^* ^–R582V^/ST, the catalytic efficiency of ND*^Dr^* ^–F219H/R582V^/ST and ND*^Dr^* ^–S480N/R582V^/ST showed a 2.07‐fold and 1.57‐fold increase, respectively, whereas other variants showed no significant improvement. Therefore, detailed kinetic characterization was focused on the ND*^Dr^* ^–F219H^/ST and ND*^Dr^* ^–S480N^/ST variants. For ND*^Dr^* ^–F219H^/ST, the *K*_m_ value (0.67 ± 0.16 mg·mL^−1^) was comparable to that of the ND*^Dr^* ^–WT^/ST, while its catalytic efficiency increased 2.73‐fold. In contrast, the ND*^Dr^* ^–S480N^/ST variant displayed a lower *K*_m_ value (0.43 ± 0.13 mg·mL^−1^), leading to a 1.98‐fold enhancement of the catalytic efficiency, primarily due to enhanced substrate binding. Guided by these results, combinatorial mutagenesis was carried out to generate a double mutant ND*^Dr^* ^–F219H/S480N^/ST and a triple mutant, designated ND*^Dr^* ^–MEc^/ST (F219H/S480N/R582V). Both variants surpassed the catalytic efficiency of the ND*^Dr^* ^–WT^/ST. Among them, ND*^Dr^* ^–MEc^/ST demonstrated the most pronounced effect, with a specific activity of 2812.94 U·mg^−1^ and a *K*_m_ of 0.27 ± 0.05 mg·mL^−1^, exhibiting a 4.08‐fold higher catalytic efficiency and a 2.66‐fold increase in specific activity compared with ND*^Dr^* ^–R582V^/ST (starting strain for the integrated mutagenesis strategy). Furthermore, it achieved a 7.48‐fold enhancement in catalytic efficiency compared to the wild‐type enzyme (ND*^Dr^* ^–WT^/ST) (Figure 6F).

**TABLE 1:** Enzyme kinetic parameters of ND*^Dr^*/ST wild type and its variants.

Enzymes	Specific activity (U·mg^−1^)	*K*_m_ (mg·mL^−1^)	*k*_cat_ (h^−1^)	*k*_cat_/*K*_m_ (mL·h^−1^·mg^−1^)
ND*^Dr^* ^–WT^/ST	775.37 ± 65.20	0.67 ± 0.22	15.22 ± 1.54	22.71 ± 2.29
ND*^Dr^* ^–R582V^/ST	1057.21 ± 44.82	0.56 ± 0.16	23.20 ± 2.20	41.59 ± 3.96
ND*^Dr^* ^–F219H/R582V^/ST	1918.07 ± 53.28	0.52 ± 0.21	44.35 ± 5.94	86.00 ± 11.52
ND*^Dr^* ^–N428H/R582V^/ST	494.79 ± 18.05	0.32 ± 0.05	7.92 ± 0.23	24.99 ± 0.88
ND*^Dr^* ^–S480N/R582V^/ST	1242.72 ± 61.69	0.36 ± 0.08	23.88 ± 1.29	65.66 ± 3.55
ND*^Dr^* ^–S506P/R582V^/ST	897.29 ± 42.11	0.34± 0.18	13.93 ± 1.32	40.79 ± 3.98
ND*^Dr^* ^–F219H^/ST	1936.46 ± 29.49	0.67 ± 0.16	41.54 ± 3.02	61.99 ± 4.50
ND*^Dr^* ^–S480N^/ST	1081.22 ± 14.14	0.43 ± 0.13	18.25 ± 1.57	45.05 ± 3.67
ND*^Dr^* ^–F219H/S480N^/ST	2083.66 ± 73.47	0.32 ± 0.06	33.70 ± 1.50	106.65 ± 4.78
ND*^Dr^* ^–MEc^/ST (F219H/S480N/R582V)	2812.94 ± 98.70	0.27 ± 0.06	45.35 ± 1.95	169.84 ± 7.31

### 2.6 Loop Mutations Enhanced ND*^Dr^* ^–MEc^/ST Performance by Reinforcing Structural Stability

Given that ND*^Dr^* ^–MEc^/ST outperformed all single and double variants in terms of catalytic efficiency, we next assessed whether these mutations also affected enzyme stability and explored the structural basis of the improved performance. Importantly, introduction of the three mutations did not alter enzyme properties (Figure S11); instead, the half‐life of ND*^Dr^* ^–MEc^/ST was prolonged from 2.5 to 12 h at 37°C, which was the optimal temperature (Figure 7A). To investigate the mechanism underlying the improved catalytic efficiency and stability, three‐dimensional structures of ND*^Dr^* ^–WT^/ST and ND*^Dr^* ^–MEc^/ST were predicted using AlphaFold, followed by 100 ns MD simulations. The average RMSD values of ND*^Dr^* ^–WT^/ST and ND*^Dr^* ^–MEc^/ST were 1.025 and 0.561 nm, respectively (Figure 7B). RMSF analysis further revealed reduced fluctuations at the mutated sites 219 (0.1171 vs. 0.1935 nm), 480 (0.1196 vs. 0.1268 nm), and 582 (0.1090 vs. 0.2098 nm) compared to ND*^Dr^* ^–WT^/ST, indicating enhanced local rigidity and structural stability (Figure 7C). To further probe conformational changes associated with enhanced activity, structural superposition and interaction network analysis were performed. In ND*^Dr^* ^–MEc^/ST, the F219H substitution in a loop region allows the histidine residue to form a hydrogen bond with K308 (2.0 Å) and participate in a stabilizing interaction network involving K308 and S303. Residue S480N forms hydrogen bonds with E409 (1.4 Å) and K452 (2.7 Å) on the adjacent α‐helix, while R582V introduced a new hydrophobic interaction with V478 (Figure 7D). These mutations collectively expanded the interaction network and reinforced structural stability. Moreover, S480N forms two additional hydrogen bonds with His652 within the substrate‐binding cleft of NST domain (Figure 7E), potentially shortening the distance to the substrate and facilitating inter‐domain substrate transfer in the artificial ND*^Dr^* ^–MEc^/ST.

**FIGURE 7:**
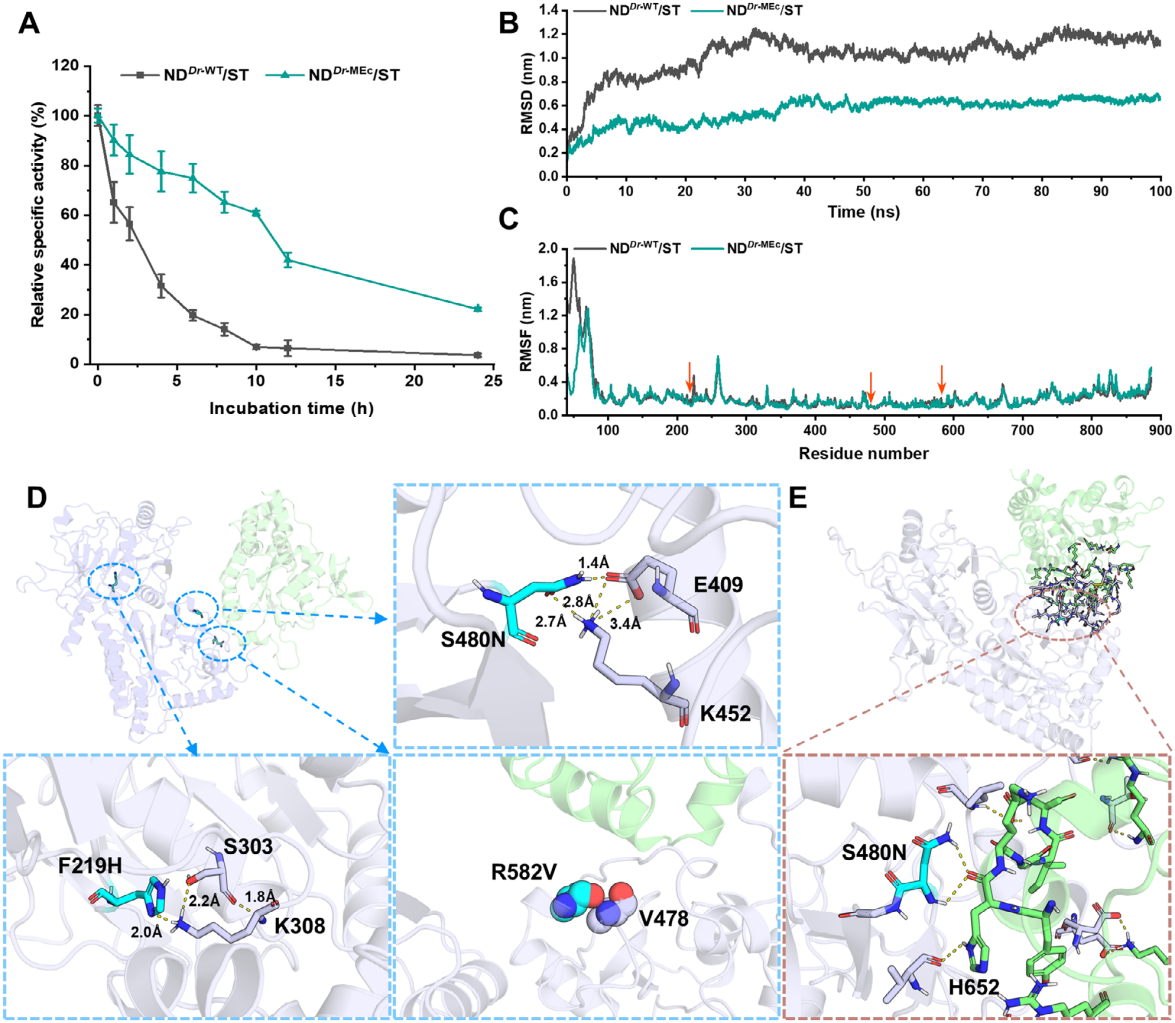
Enzymatic property analysis and structure comparison. (A) Stability analysis of ND*^Dr^* ^–WT^/ST and ND*^Dr^* ^–MEc^/ST at 37°C. (B) RMSD and (C) RMSF of ND*^Dr^* ^–WT^/ST and ND*^Dr^* ^–MEc^/ST. (D) Change in interaction forces between the ND*^Dr^* ^–WT^/ST and ND*^Dr^* ^–MEc^/ST. (E) Close‐up view of the interactions between the ND*^Dr^* and NST domains in the ND*^Dr^* ^–MEc^/ST. Light‐blue color represents the ND*^Dr^* domain, while green color represents the NST domain. The yellow dashed line indicates the formed hydrogen bond.

### 2.7 Efficient *N*‐Sulfation of Heparosan With the Engineered ND*^Dr^* ^–MEc^/ST

The catalytic efficiency of the artificial bifunctional enzyme toward heparosan serves as a key indicator for evaluating its functional performance. Given that the pentasaccharide sulfation pattern constitutes the key antithrombin‐binding motif, the *N*‐sulfation activities of the ND*^Dr^* ^–WT^/ST and the variant ND*^Dr^* ^–MEc^/ST toward both structurally defined heparosan oligosaccharides and long‐chain heparosan were compared. As shown in Figure 8A, both enzymes exhibited catalytic activity toward heparosan pentasaccharide (HN5^NN^). In contrast to the ND*^Dr^* ^–WT^/ST, which showed limited catalytic efficiency, the ND*^Dr^* ^–MEc^/ST generated a clearly detectable *N*‐sulfated HN5^NN^ (HN5^NN^‐NS), as evidenced by a distinct mass peak at *m/z* 1016.2 corresponding to [M–H]^−^. Following depolymerization of enzymatic products into disaccharides for sulfation analysis, HN5^NN^‐NS generated by ND*^Dr^* ^–MEc^/ST exhibited an *N*‐sulfation degree of approximately 11% (Figure 8B). With increasing chain length, the *N*‐sulfation efficiency of both ND*^Dr^* ^–WT^/ST and the ND*^Dr^* ^–MEc^/ST variant progressively improved, reaching 19.4% and 35.7% for heparosan octasaccharide (HN8^AN^), respectively.

**FIGURE 8:**
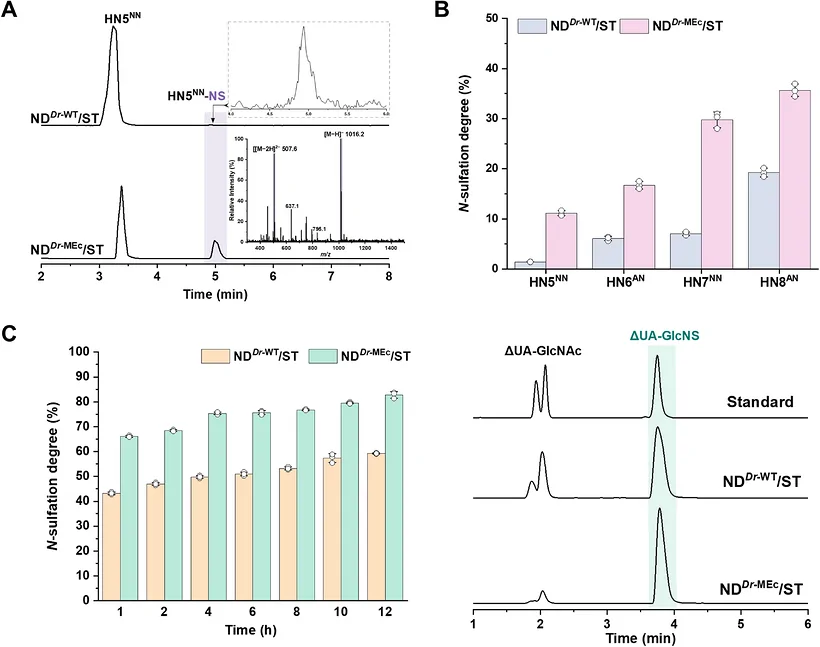
*N*‐sulfation analysis of catalytic products. (A) UPLC chromatograms and mass spectra of HN5^NN^ and enzymatically produced HN5^NN^‐NS. (B) Comparison of sulfation levels in products catalyzed by ND*^Dr^* ^–WT^/ST and ND*^Dr^* ^–MEc^/ST using substrates of varying chain lengths. (HN7^NN^, heparosan heptasaccharide) (C) Comparison of sulfation levels in products generated by ND*^Dr^* ^–WT^/ST and ND*^Dr^* ^–MEc^/ST, validated by UPLC chromatograms of *N‐*sulfated heparosan disaccharides.

For long‐chain heparosan, the ND*^Dr^* ^–WT^/ST achieved an *N*‐sulfation degree of approximately 59.2% (Figure 8C). Although this represents a significant improvement relative to the original artificial bifunctional enzyme (Figure 2F), the sulfation level remains below the threshold required for producing biologically active heparin, which typically demands higher *N*‐sulfation for anticoagulant functionality. In contrast, the variant ND*^Dr^* ^–MEc^/ST exhibited a markedly accelerated and enhanced catalytic performance, as evidenced by the emergence of a distinct product peak. Within just 1 h of reaction, ND*^Dr^* ^–MEc^/ST reached an *N*‐sulfation degree of 66.1%, slightly higher than the maximum conversion achieved by the ND*^Dr^* ^–WT^/ST after 12 h. As the reaction progressed, the *N*‐sulfation degree further increased to 82.8% (Figure 8C). This dramatic enhancement underscores the improved substrate recognition and catalytic efficiency of ND*^Dr^* ^–MEc^/ST. The apparent molecular weight of the catalytic product was comparable to that of heparosan (Figure S12). The corresponding nuclear magnetic resonance spectra of heparosan and *N*‐sulfated heparosan are shown in Figures S13‐S14. Subsequent analysis of the depolymerized *N*‐sulfated heparosan confirmed the presence of ‐GlcA‐GlcNS‐ repeating units, consistent with those generated by *Homo sapiens* NDST expressed in *Komagataella phaffii* (Figure S15A–C), and further treatment with downstream heparin biosynthetic enzymes (C5‐epimerase and 2‐*O*‐sulfotransferase) demonstrated that the enzymatically generated *N*‐sulfated heparosan could serve as a competent substrate for heparin biosynthesis (Figure S15D,E). Taken together, these findings demonstrate that rational engineering successfully overcame the expression and activity bottlenecks of NDST in *E. coli*, and established ND*^Dr^* ^–MEc^/ST as a promising and efficient biocatalyst for the synthesis of *N*‐sulfated heparosan.

## 3 Discussion

Heparin biosynthesis requires a tightly coordinated cascade of enzymatic modifications, in which NDST plays the initiating and rate‐limiting role by catalyzing both *N*‐deacetylation and *N*‐sulfation of heparosan [32, 33]. However, the functional expression of NDST in prokaryotic systems has long remained a major bottleneck, while eukaryotic systems (e.g., insect cells) [34] generally involve relatively high production costs, complex culture conditions, and limited scalability for biomanufacturing applications. Alternative chemical or semi‐synthetic approaches—such as NaOH‐mediated deacetylation or unnatural monosaccharide substitution—either disrupt the natural structure of heparin or hinder scalability [17, 21]. Here, we engineered and expressed an artificial NDST in *E. coli*, enabling efficient biosynthesis of *N*‐sulfated heparosan.

A critical barrier resides in the NDase domain, which determines the activity of NDST. Heparosan‐specific NDases share a conserved metal‐assisted acid/base mechanism with the polysaccharide NDases of the CE family, characterized by a canonical Asp‐His‐His motif that coordinates a catalytic metal ion [20, 35]. Since natural CE family NDases typically act on acetyl‐rich polysaccharides, we reasoned that they could potentially accommodate heparosan. Screening revealed that while ND*^Mr^* and other enzymes share similar catalytic pattern with ND*^Dr—^* such as a preference for long‐chain substrates and directional catalysis from the non‐reducing to the reducing end [36, 37, 38]—they displayed weak activity toward heparosan and failed to enhance bifunctional constructs despite linker optimization (Figure 2 and Figure S7). These results suggest that the enzymes identified in this study likely possess substrate promiscuity, consistent with their evolutionary relationship to polysaccharide deacetylases acting on diverse acetylated polysaccharides. This functional diversity highlights their potential for broader applications in polysaccharide engineering, including offering new opportunities for the modification of other glycosaminoglycans, and provides a useful reference for understanding substrate recognition mechanisms in heparosan‐specific NDases. In contrast, ND*^Dr^* proved superior in supporting bifunctional enzyme assembly—the fusion of the NDase and NST domains during evolution, like other bifunctional enzymes, may have been driven by selective pressure to enhance catalytic activity [39], particularly through cooperative interaction with the NST domain.

More importantly, beyond its established role in supporting cooperative bifunctional enzyme function [18], we found that the NST domain plays a pivotal role in mediating inter‐domain communication and modulating the catalytic conformation of NDase. Although in some CE4 family NDases, NST interactions may sterically interfere with substrate binding (Figures S8 and S9), our results demonstrate that in ND*^Dr^*‐based constructs, NST actually enhances ND*^Dr^* activity, even in the absence of the sulfonate group PAPS that normally required for NST catalyzation (Figure 3). This observation supports the model of functional cooperativity in full‐length ND*^Dr^*‐RL‐ST, whereby the NST domain stabilizes the ND*^Dr^* loop in an open conformation, thereby facilitating ND*^Dr^* active‐site formation and supporting its catalytic function (Figure 4C) [20].

Sequence‐guided engineering offers a powerful approach for enhancing enzyme activity by stabilizing catalytically relevant conformations and reinforcing active‐site architecture [40, 41, 42]. Complementary MD simulations and DCCM analysis reveal intramolecular correlations between residues and have been instrumental in elucidating allosteric regulation mechanisms in enzymes, providing an effective approach for identifying candidate mutation sites [43]. Multiple sequence alignment then provides statistical amino acid frequencies, highlighting conserved but flexible positions and indicating which residue substitutions can be introduced through mutagenesis [44, 45, 46]. By integrating DCCM analysis based on MD simulations with consensus analysis and mutation‐effect prediction, we established a rational engineering workflow for site selection. This strategy led to the construction of the engineered variant ND*^Dr^* ^–MEc^/ST, which exhibited a 7.48‐fold increase in catalytic efficiency together with a markedly prolonged half‐life relative to the wild type enzyme (Figures 6F and 7A). Structural and interaction analyses revealed that the introduced mutations enhanced inter‐domain communication, reinforced local structural rigidity, and preserved the conformational flexibility required for catalysis, thereby collectively improving both activity and stability (Figure 7D,E). Building on these insights, the combined strategy integrates dynamic‐ and sequence‐guided mutagenesis, enabling efficient identification of candidate mutation sites and synergistic modulation of enzyme function, providing a general framework for the rational design of high‐performance biocatalysts.

The recent thrust towards development has focused on heparin biotransformation in *E. coli* [47, 48]. The conversion analysis revealed that the ND*^Dr^* ^–MEc^/ST achieved the final sulfation level of the wild‐type enzyme within a markedly shorter reaction period (Figure 8). Compared with chemical deacetylation, which often results in heterogeneous products and backbone degradation under harsh conditions [17], the enzymatic approach provides higher structural uniformity with reduced polysaccharide backbone damage. Such properties may be attributed to the intrinsic regioselectivity and substrate specificity of the bifunctional enzyme, enabling controlled modification while preserving its integrity (Figure S12). Furthermore, ND*^Dr^* ^–MEc^/ST catalyzed the *N*‐sulfation of heparosan to a final 82.8% conversion, surpassing the threshold required for biologically active heparin synthesis (≥80%) [49]. The resulting product, consistent with those generated by NDST expressed in *K. phaffii*, forms clusters of ‐GlcA‐GlcNS‐ repeating units as previously reported [18, 32] and functions as an effective substrate for subsequent catalysis (Figure S15). These findings underscore the importance of NDase screening and characterization for constructing functional artificial NDSTs, and contribute to the growing evidence supporting *E. coli* as a viable microbial chassis for enzymatic heparin biosynthesis. Beyond heparin, this work provides a blueprint for engineering artificial multifunctional enzymes capable of orchestrating complex polysaccharide modifications, offering broader applications in glycoengineering and biomanufacturing.

## 4 Conclusion

In this study, we systematically screened and characterized multiple *N*‐deacetylases (NDases) targeting heparosan in *E. coli*, by coupling NDase activity with *N*‐sulfotransferase (NST) through cascade reactions, we confirmed the ability of these enzymes to produce *N*‐sulfated heparosan, providing a foundational understanding of substrate specificity and catalytic potential. Inspired by the natural fusion of deacetylases and sulfotransferases, we engineered artificial NDSTs by linking NDases and the NST domain with a rigid linker, identifying ND*^Dr^* as a standout candidate with superior catalytic performance in artificial bifunctional constructs. Further rational engineering and combinatorial design produced the variant ND*^Dr^* ^–MEc^/ST, which demonstrated markedly improved catalytic efficiency, substrate recognition, and structural stability. Kinetic analysis and molecular dynamics simulations revealed that the mutations optimized the active site conformation and interaction network, contributing to both enhanced activity and prolonged enzyme half‐life. Functionally, ND*^Dr^* ^–MEc^/ST achieved rapid and high‐degree sulfation of *N*‐sulfated heparosan, significantly surpassing the performance of the wild‐type enzyme. Collectively, these results highlight the critical role of NDases selection, NST adaption and comprehensive characterization in designing functional bifunctional enzymes, provide a robust strategy for optimizing artificial NDSTs, and provide a promising platform for the scalable biosynthesis of biologically active *N*‐sulfated heparosan, advancing the enzymatic production of heparin.

## 5 Experimental Section

### 5.1 Plasmid and Strain Construction

The plasmids and strains used in this study are summarized in Table S1, and the designed primers are listed in Table S2. *E. coli* JM109 was used for recombinant plasmid construction. *E. coli* Rosetta (DE3) was employed for protein expression.

Codon‐optimized NDase genes were synthesized by GENEWIZ, Inc. (Suzhou, China) and cloned into the multiple cloning sites of the plasmid pET32a (+) (Novagen, Germany). The maltose‐binding protein (MBP) gene was cloned from the genome of *E. coli* MG1655 using the primer pair MBP‐F/MBP‐R, and fused to the N‐terminus of the eukaryote‐derived NDases via G4S linker through seamless cloning to construct the recombinant plasmids. The NST gene was obtained from a previous publication [22] and fused to the C‐terminus of the active NDases via a rigid linker (EAAAK) to construct artificial bifunctional enzymes. The engineered enzyme variants were constructed by PCR using plasmid pET32a‐ND*^Dr^*‐RL‐ST as the template, which contains the gene‐encoding ND*^Dr^*‐RL‐ST. The amino acid sequence is provided in Table S3 of the supporting information. All constructs were sequenced by the GENEWIZ, Inc.

### 5.2 Protein Expression and Purification

Common reagents were purchased from Sinopharm Chemical Reagent Co., Ltd. (Beijing, China). Luria–Bertani (LB) medium (10 g·L^−1^ tryptone, 5 g·L^−1^ yeast extract, 10 g·L^−1^ NaCl) was used for plasmid propagation and cell inoculation. Ampicillin (100 µg·mL^−1^) and chloramphenicol (50 µg·mL^−1^) were added to provide selective pressure. The recombinant strains were pre‐cultured in LB medium grown for 10 h at 37°C. Then the strains were transferred to Terrific Broth (TB) medium (12 g·L^−1^ tryptone, 24 g·L^−1^ yeast extract, 5 g·L^−1^ glycerol, 2.54 g·L^−1^ K_2_HPO_4_, 2.31 g·L^−1^ KH_2_PO_4_) with 1% (*v/v*) inoculum size and then grown at 37°C. When the OD_600_ reached 0.6–0.8, a final concentration of 0.5 mM IPTG was added for induction and continued to cultivate at 25°C for another 16–20 h.

The cells were collected and subjected to ultrasonication followed by centrifugation at 4°C, then the supernatants were loaded onto the HisTrap FF (Cytiva, USA) through AKTA start 25 (GE Healthcare, USA) and proteins were eluted in elution buffer (20 mM Tris‐HCl, pH 7.5, 500 mM imidazole, and 500 mM NaCl). Purified protein solution was desalted using HiTrap desalting column (Cytiva, USA) with washing buffer (20 mM Tris‐HCl, pH 7.5). Protein concentration was determined by Bradford protein assay kit (Beyotime Biotechnology Co., Ltd., Shanghai, China) and the purity was analyzed by sodium dodecyl sulfate–polyacrylamide gel electrophoresis (SDS‐PAGE).

### 5.3 Deacetylation of Chitin Oligosaccharides

The deacetylation reaction mixture contained 0.1 g·L^−1^ triacetylchitotriose or pentaacetylchitopentaose (Yuanye Bio‐Technology Co., Ltd, Shanghai, China), an appropriate amount of NDases, with CoCl_2_ (0.01–1 mM) [28, 37], MnCl_2_ (0.5 mM) [29], or CaCl_2_ (10 mM) used depending on the enzyme. The reactions were carried out at 37°C for 12 h and terminated by heating at 100°C for 10 min. The resulting mixtures were then centrifuged at 12,000 rpm for 10 min, and the supernatant was filtered through a 0.22 µm hydrophilic membrane for analysis.

### 5.4 Enzyme Activity Assay

The cascade reaction solution contained 1.0 g·L^−1^ heparosan (prepared in our laboratory), an appropriate amount of NDase supernatant, and one of the following metal ions: CoCl_2_ (0.01–10 mM), MnCl_2_ (0.5 mM) or CaCl_2_ (10 mM). The reaction was incubated at 37°C for 48 h and terminated by heating at 100°C for 10 min. Subsequently, 50 mM *para*‐nitrophenyl sulfate (pNPS), 0.5 mM 3′‐phosphoadenosine‐5′‐phosphate (PAP), 2 mg·mL^−1^ aryl sulfotransferase IV, 20% (*v/v*) glycerol, and 2 mg·mL^−1^ NST were supplied. The total reaction volume was made up to 1 mL with 20 mM Tris‐HCl buffer (pH 7.5), followed by a second incubated at 37°C for 12 h.

Activity assays of artificial bifunctional enzyme were performed in a total volume of 1 mL, containing 1.0 g·L^−1^ heparosan, 50 mM pNPS, 0.5 mM PAP, 2 mg·mL^−1^ aryl sulfotransferase IV, 20% (*v/v*) glycerol, 10 mM CaCl_2_, and 500 µL of the artificial bifunctional enzyme supernatant. The reaction mixture was incubated for 12 h at 37°C. One unit of enzyme activity was defined as the amount of enzyme that catalyzes the conversion of 1 µmol of pNPS to *para*‐nitrophenol (pNP) per hour under the assay conditions at 37°C. The production of pNP was measured by monitoring the absorbance at wavelength 400 nm.

### 5.5 Characterization of ND*^Dr^*/ST and Selected Variants

Kinetic parameters were determined by measuring enzyme activities at various substrates concentrations (0–0.8 mg·mL^−1^). Data were fitted to the Michaelis‐Menten equation and analyzed using a double reciprocal plot using OriginPro (Learning Edition, OriginLab Corporation, USA). The optimal temperature was determined by measuring enzyme activity at temperatures ranging from 30–50°C, while the optimum pH was determined at 37°C over a pH range of 5.0–10.6. Enzyme stability was determined by pre‐incubating the enzyme at specific temperatures for different durations, and the residual activity was measured under standard assay conditions as described above. All experiments were performed in triplicate independently and the results are presented as the mean values ± standard deviation.

### 5.6 Qualitative Analysis of Enzymatic Products

The catalytic system includes 1.0 g·L^−1^ heparosan or 0.2 g·L^−1^ heparosan oligosaccharides (prepared in our laboratory [38]), PAPS (substrate:PAPS molar ratio = 1:2, prepared in our laboratory [11, 50]), 10 mM CaCl_2_, and either the engineered bifunctional enzyme or *Homo sapiens* NDST expressed in *K. phaffii* [38, 51] at 0.2 g·L^−1^. Reactions were performed at 37°C and terminated by heating at 100°C for 10 min. Subsequent modification reactions were conducted using 0.2 g·L^−1^ *N*‐sulfated heparosan, PAPS at a 2‐fold molar ratio relative to the substrate, and C5‐epimerase/2‐*O*‐sulfotransferase (prepared in our laboratory [52]) under the same conditions. The products were digested with heparinase III (purified in our laboratory) at 37°C for 6–12 h, resulting in partial to complete depolymerization. After centrifugation (12,000 rpm, 10 min), the supernatant was filtered through a 0.22 µm hydrophilic membrane to obtain disaccharides for analysis.

HPLC was performed according to the method described in reference [15]. The chromatographic separation was performed using an Spherisorb SAX column (4.0 × 250 mm, 5.0 µm, Waters) maintained at 40°C, with a flow rate of 1 mL·min^−1^. The mobile phase consisted of Phase A: 1.8 mM sodium dihydrogen phosphate solution, and Phase B: 1.8 mM sodium dihydrogen phosphate solution and 1 M sodium perchlorate solution. The gradient program was as follows: 0–20 min, linear increase of mobile phase B from 0% to 100%. The peak area was measured by monitoring the absorbance at wavelength 232 nm. The *N*‐sulfation degree was determined by calculating the proportion of *N*‐sulfated heparosan disaccharide peak area relative to the total disaccharide peak area based on chromatographic peak area integration.

UPLC was carried out at 45°C on a XSelect CSH C18 column (1.7 µm, 2.1 × 50 mm) from Waters. Eluent A consisted of 0.1 M formic acid and eluent B was acetonitrile. The mass spectra of oligosaccharides and their sulfated derivatives were acquired on MALDI SYNAPT Q‐TOF MS (Waters) in negative ionization mode, the cone voltage was 20 V, the detection voltage was 3.0 kV, the capillary voltage was 3.0 kV, the source block temperature of 100°C and the desolation temperature of 400°C to obtain spectra in the range of 20–1500 *m/z* at a collision energy was 6/20 V. The data were analyzed using MassLynx software (Waters).

### 5.7 Molecular Weight Determination

The heparosan and its catalyzed products were analyzed by gel permeation chromatography‐high performance liquid chromatography (GPC‐HPLC). The GPC‐HPLC was used with an Ultrahydrogel linear column (7.8 mm × 300 mm i.d., Waters), eluted with 0.1 M sodium nitrate at 40°C at a flow rate of 0.6 mL·min^−1^ and a run time of 35 min. Calibration curve used for GPC analysis: log(Mw) = 14.0 − 0.554x. (R^2^ = 0.9978)

### 5.8 Nuclear Magnetic Resonance Analysis

Heparosan and its enzymatic products were purified using dialysis with a 10 kDa MWCO membrane, followed by lyophilization and dissolution in 500 µL of D_2_O (99.9%, Sigma‐Aldrich) for further analysis. Nuclear magnetic resonance (NMR) spectra were recorded on a Bruker Advance NEO spectrometer at 600 MHz. The data were processed and analyzed using MestReNova software (Version 15.0.1, Mestrelab Research).

### 5.9 Molecular Dynamics Simulations and Dynamic Cross‐Correlation Matrices Analysis

The initial structural models of ND*^Dr^* ^–WT^/ST and ND*^Dr^* ^–MEc^/ST were generated by AlphaFold based on PDB structure 8CCY. MD simulations were performed at 310.15 K using the GROMACS [53]. The protein was placed in a cubic box and solvated with water, and the charge of the system was neutralized by adding Na^+^ and Cl^–^. The system had been energy minimized and NVT (canonical ensemble) and NPT (constant pressure, constant temperature) equilibrated before the simulations. The final conformations were then used for structural analysis. To quantify the correlation between the residues based on the direction and magnitude of their movement, DCCM analysis was performed using the backbone Cα atoms extracted from the MD simulation trajectories. DCCM values range from −1.00, indicating fully negative correlations, to +1.00, indicating fully positive correlations.

### 5.10 In Silico Analysis

Correlated sequences were acquired from the UniProt (https://www.uniprot.org/) and CAZy Database (https://www.cazy.org/), the data visualization was performed using Cytoscape [54]. Multiple sequence alignment was conducted with ClustalW (https://www.genome.jp/tools‐bin/) and visualized using Weblogo [55]. Maximum‐likelihood phylogenetic tree was constructed with the IQ‐Tree [56] and visualized with iTOL [57]. Mutagenesis design was performed with EVmutation [58]. ProteinTools server [59] and Cluspro [60] were used to analyze the interaction forces. Pymol was employed for protein structures visualization and image processing.

## Author Contributions

**Xintong Xi**: Writing – original draft, Visualization, Methodology, Data curation, Conceptualization. **Ruirui Xu**: Writing – review & editing, Conceptualization, Data curation. **Daoan Wang**: Writing – review & editing, Data curation. **Guobin Yin**: Methodology, Data curation. **Xinjing Wang**: Methodology, Data curation. **Haiqin Chen**: Supervision, Conceptualization. **Guocheng Du**: Supervision, Conceptualization. **Jian Chen**: Funding acquisition, Conceptualization. **Zhen Kang**: Writing – review & editing, Funding acquisition.

## Conflicts of Interest

The authors declare no conflicts of interest.

## Supporting information




**Supporting File**: advs76499‐sup‐0001‐SuppMat.docx.

## Data Availability

Data will be made available on request.
