# Supplementary material for: Design and Engineering of an Artificial Bifunctional N‐Deacetylase/N‐Sulfotransferase for the Biosynthesis of N‐Sulfated Heparosan
Source: Adv Sci (Weinh). 2026 Jul 10;13(55):e76499. doi: 10.1002/advs.76499 (PMC13353177; doi:10.1002/advs.76499)
Supplement: Supplementary file 1 [file ADVS-13-e76499-s001.docx]

Supporting Information

Design and Engineering of an Artificial Bifunctional *N*-Deacetylase/*N*-Sulfotransferase for the Biosynthesis of *N*-Sulfated Heparosan

*Xintong Xi^1,2,3^*, *Ruirui Xu^1,3,4^*, *Daoan Wang^1,3,4^*, *Guobin Yin^1,3,4^*, *Xinjing Wang^1,3,4^*, *Haiqin Chen^2^*, *Guocheng Du^1,3,4^*, *Jian Chen^1,3,4^*, *Zhen Kang^1,3,4*^*

^1^The Key Laboratory of Carbohydrate Chemistry and Biotechnology, Ministry of Education, Jiangnan University, Wuxi, 214122, China

^2^School of Food Science and Technology, Jiangnan University, Wuxi, 214122, China ^3^The Science Center for Future Foods, Jiangnan University, Wuxi, 214122, China

^4^Jiangsu Province Basic Research Center for Synthetic Biology, Jiangnan University, Wuxi, 214122, China

*Corresponding author

E-mail for Zhen Kang: [zkang@jiangnan.edu.cn](mailto:zkang@jiangnan.edu.cn)

Phone: +86-510-85918307, Fax: +86-510-85918309 (Z. Kang)

Table S1. Strains and plasmids used in this study

| Strains or plasmids | Characteristics | Source |
| --- | --- | --- |
| *E. coli* strains |  |  |
| *E. coli* JM109 | Cloning host | Invitrogen |
| *E. coli* Rosetta (DE3) | Expression host, Cm^R^ | Invitrogen |
| Plasmids |  |  |
| pET-32a (+) | Expression vector, Amp^R^ | Novagen |
| pET32a-ND*^Sp^* | pET-32a (+) carrying *N*-deacetylase gene of *Streptococcus pneumoniae* (UniProtKB—A0A0H2ZN69), Amp^R^ | This work |
| pET32a-ND*^Mr^* | pET-32a (+) carrying MBP gene and *N*-deacetylase gene of *Mucor rouxii* (UniProtKB—P50325) with flexible linker, Amp^R^ | This work |
| pET32a-ND*^Ar^* | pET-32a (+) carrying MBP gene and *N*-deacetylase gene of *Arthrobacter sp* (UniParc—UPI000B8BB35C) with flexible linker, Amp^R^ | This work |
| pET32a-ND*^Sl^* | pET-32a (+) carrying MBP gene and *N*-deacetylase gene of *Streptomyces lividans* (UniProtKB—A0ABM5R7G1) with flexible linker, Amp^R^ | This work |
| pET32a-ND*^Af^* | pET-32a (+) carrying MBP gene and *N*-deacetylase gene of *Aspergillus fumigatus* (UniProtKB—Q4WX15) with flexible linker, Amp^R^ | This work |
| pET32a-ND*^Hs^* | pET-32a (+) carrying MBP gene and *N*-deacetylase gene of *Homo sapiens* (UniProtKB—P52848) with flexible linker, Amp^R^ | This work |
| pET32a-ND*^Dr^* | pET-32a (+) carrying MBP gene and *N*-deacetylase gene of *Danio rerio* (UniProtKB—A0A0R4IR84) with flexible linke, Amp^R^ | This work |
| pET32a-ND*^Pv^* | pET-32a (+) carrying MBP gene and *N*-deacetylase gene of *Penaeus vannamei* (UniParc—UPI000F662199) with flexible linker, Amp^R^ | This work |
| pET32a-ND*^Mr^*-RL-ST | pET32a-ND*^Mr^* carrying NST gene with rigid linker, Amp^R^ | This work |
| pET32a-ND*^Ar^*- RL-ST | pET32a-ND*^Ar^* carrying NST gene with rigid linker, Amp^R^ | This work |
| pET32a-ND*^Sl^*- RL-ST | pET32a-ND*^Sl^* carrying NST gene with rigid linker, Amp^R^ | This work |
| pET32a-ND*^Af^*- RL-ST | pET32a-ND*^Af^* carrying NST gene with rigid linker, Amp^R^ | This work |
| pET32a-ND*^Dr^*-RL-ST | pET32a-ND*^Dr^* carrying NST gene with rigid linker, Amp^R^ | This work |
| pET32a-ND*^Pv^*-RL-ST | pET32a-ND*^Pv^* carrying NST gene with rigid linker, Amp^R^ | This work |

Table S2. Primers used in this study

| Name | Sequences (5' - 3') |
| --- | --- |
| vector-F | TAAAAGCTTGCGGCCGCACTCGAGC |
| vector-R | GGATCCGATATCAGCCATGGCCTTGTCGT |
| MBP-F | ATGGCTGATATCGGATCCATGAAAATCGAAGAAGGTAAACTGGT |
| MBP-R | GCTGCCGCCGCCGCCAGTCTGCGCGTCTTTCAGG |
| ND*^Sp^*-F | GCCATGGCTGATATCGGATCCATGAGCAATTGCATCATCGAAATCAT |
| ND*^Sp^*-R | GTGCGGCCGCAAGCTTTTATTCATCGCGGCTGTAGTACAGT |
| ND*^Mr^*-F | GGCGGCGGCGGCAGCGATACCAGCGCCAATTACTGGCAGAG |
| ND*^Mr^*-R | GTGCGGCCGCAAGCTTTTAAACATTGGTCCATTCGTAATCTTCGAAG |
| ND*^Ar^*-F | CAGACTGGCGGCGGCGGCAGCGCTGGACAACCTGAACCAGTAGC |
| ND*^Ar^*-R | AGTGCGGCCGCAAGCTTTTACGGGTTCGTCTTAAAACGATGGT |
| ND*^Sl^*-F | ACTGGCGGCGGCGGCAGCGCTGCATGTAATGGATATGTAGGGCT |
| ND*^Sl^*-R | AGTGCGGCCGCAAGCTTTTAGCCGGCGGTACAGCTGACAGATG |
| ND*^Af^*-F | ACTGGCGGCGGCGGCAGCCACGGCAGCGTTCTGAGCAATAT |
| ND*^Af^*-R | AGTGCGGCCGCAAGCTTTTATTCCAGGGCGATCGGGGTGCTCA |
| ND*^Dr^* -F | GGCGGCGGCGGCAGCGGCGTAAAGCGCGAACTTGAACC |
| ND*^Dr^* -R | GTGCGGCCGCAAGCTTTTATTTAGGAAAACGATCACACGTCTTTTCCT |
| ND*^Hs^* -F | GGCGGCGGCGGCAGCGGATGGAAAAGGGGGCTAGAACC |
| ND*^Hs^* -R | CTCGAGTGCGGCCGCAAGCTTTTATTTTGGGAAGCGATCACAGGTCTTCT |
| ND*^Dr^* -ST-R | ATCACACGTCTTTTCCTTCGACCAGATATCCT |
| ND*^Dr^* -ST-F | TCGAAGGAAAAGACGTGTGATAGATTCCCTAAGTTGTTGATCAT |
| ND*^Mr^*-RL-ST-F | GAATGGACCAATGTTGAAGCGGCGGCGAAAAGATTCCCTAAGTTGTTGATCAT |
| ND*^Ar^* -RL-ST-F | TTTAAGACGAACCCGGAAGCGGCGGCGAAAAGATTCCCTAAGTTGTTGATCAT |
| ND*^Sl^* -RL-ST-F | AGCTGTACCGCCGGCGAAGCGGCGGCGAAAAGATTCCCTAAGTTGTTGATCAT |
| ND*^Af^* -RL-ST-F | CCGATCGCCCTGGAAGAAGCGGCGGCGAAAAGATTCCCTAAGTTGTTGATCAT |
| ND*^Dr^* -RL-ST-F | GAATGGACCAATGTTGAAGCGGCGGCGAAAAGATTCCCTAAGTTGTTGATCAT |
| ND*^Pv^* -RL-ST-F | ATCTGGTCCAAGAACGAAGCGGCGGCGAAAAGATTCCCTAAGTTGTTGATCAT |
| G6-ST-F | GAATGGACCAATGTTGGTGGCGGTGGCGGCGGTAGATTCCCTAAGTTGTTGATCAT |
| G4S-ST-F | GAATGGACCAATGTTGGCGGTGGTGGTAGCAGATTCCCTAAGTTGTTGATCAT |
| ST-R | TGCGGCCGCAAGCTTTTATCTAGTATTTTGCAAATCCTCTCTCAACC |
| Nolinker-MBP-R | AGTCTGCGCGTCTTTCAGGGCTTCAT |
| MBP-ND*^Dr^* -F | GGCGTAAAGCGCGAACTTGAACCAGC |
| MBP-G4S2-R | AGAACCACCACCACCTGAACCACCGCCACCAGTCTGCGCGTCTTTCAGGG |
| MBP-EAAAK-R | TTTGGCGGCCGCTTCAGTCTGCGCGTCTTTCAGGG |
| MBP-G6-R | GCCTCCACCGCCGCCGCCAGTCTGCGCGTCTTTCAGGG |
| MBP-PT4-R | GGTCGGGGTCGGGGTCGGGGTCGGAGTCTGCGCGTCTTTCAGGG |
| MBP-PT2-R | GGTCGGGGTCGGAGTCTGCGCGTCTTTCAGGG |
| D110E-F | AGTCAGTTGGGCCAG**GAA**ATTGTCGCCATCTT |
| D110/I111-R | CTGGCCCAACTGACTGTACAGTGATT |
| I111V-F | AGTCAGTTGGGCCAGGAT**GTT**GTCGCCATCTT |
| G118S-F | GCCATCTTGGAGTCC**AGC**CGCTTCCGCTATCAAAC |
| G118-R | GGACTCCAAGATGGCGACAATATCCTGGC |
| Q189L-F | AATGAAAATTCTTTG**CTG**TCTGCTCAGTTGAAAGG |
| Q189-R | CAAAGAATTTTCATTTGCTTTGAAGAAAC |
| F219H-F | AAATCGCCACTTTTA**CAT**ATCACCAAGGCGCG |
| F219-R | TAAAAGTGGCGATTTGGGGTTGACGGAGC |
| F219Y-F | AAATCGCCACTTTTA**TAT**ATCACCAAGGCGCG |
| K222R-F | CTTTTATTTATCACC**CGT**GCGCGCGAAGTAGAGCATGG |
| K222-R224-R | GGTGATAAATAAAAGTGGCGATTTGGGGT |
| A223P-F | CTTTTATTTATCACCAAG**CCG**CGCGAAGTAGAGCATGG |
| A223S-F | CTTTTATTTATCACCAAG**AGC**CGCGAAGTAGAGCATGG |
| R224F-F | CTTTTATTTATCACCAAGGCG**GGT**GAAGTAGAGCATGG |
| R224S-F | CTTTTATTTATCACCAAGGCG**AGC**GAAGTAGAGCATGG |
| E227/H228-R | TACTTCGCGCGCCTTGGTGATAAATAAAAGTG |
| E227L-F | AAGGCGCGCGAAGTA**CTG**CATGGCCCTCTGCC |
| H228K-F | AAGGCGCGCGAAGTAGAG**AAA**GGCCCTCTGCCTGG |
| H228P-F | AAGGCGCGCGAAGTAGAG**CCG**GGCCCTCTGCCTGG |
| P230-R | GCCATGCTCTACTTCGCGCGCCTTGG |
| P230V-F | GAAGTAGAGCATGGC**GTT**CTGCCTGGCGATGATTG |
| D234E-F | GGCCCTCTGCCTGGC**GAA**GATTGGACCGT |
| D234-R | GCCAGGCAGAGGGCCATGCTCTACTT |
| T244S-F | TTTCAATCCAATCAT**AGC**ACCTATGAGCCTGT |
| T244-R | ATGATTGGATTGAAAGACGGTCCAATCATCGC |
| G254T-F | CCTGTGCTGCTGGCGCGC**ACC**CGTTCCACTGAAGC |
| G254L-F | CCTGTGCTGCTGGCGCGC**CTG**CGTTCCACTGAAGC |
| R255K-F | CCTGTGCTGCTGGCGCGCGGC**AAA**TCCACTGAAGC |
| R255Q-F | CCTGTGCTGCTGGCGCGCGGC**CAG**TCCACTGAAGC |
| G254/R255-R | CGCCAGCAGCACAGGCTCATAGGTGGTAT |
| A259S-F | GGCCGTTCCACTGAA**AGC**AGTGGCGCCCCAGGCCC |
| A259/S260-R | TTCAGTGGAACGGCCGCGCGCCAGCAGCACAG |
| S260I-F | GGCCGTTCCACTGAAGCA**ATT**GGCGCCCCAGGCCC |
| S260V-F | GGCCGTTCCACTGAAGCA**GTT**GGCGCCCCAGGCCC |
| A269S-F | GGCCCATTACATGCC**AGC**GTTGTTCAGGACTT |
| A269T-F | GGCCCATTACATGCC**ACC**GTTGTTCAGGACTT |
| A269-R | GGCATGTAATGGGCCTGGGGCGCCACTTG |
| L298F-F | CTTCACAAGTTAGTG**TTT**GTTGACGCGGTGTC |
| L298-R | CACTAACTTGTGAAGCCAAAAGTTAAGAT |
| A313G-F | AAGCGCTTATCGTTA**GGT**TTGGAACGTTATAT |
| A313P-F | AAGCGCTTATCGTTA**CCG**TTGGAACGTTATAT |
| A313S-F | AAGCGCTTATCGTTA**AGC**TTGGAACGTTATAT |
| A313-R | TAACGATAAGCGCTTGCCTGTAAGGAACG |
| N351T-F | CAAAATGAATTGCGT**ACC**CACGTACCGAATTT |
| N351-R | ACGCAATTCATTTTGTGTTTCAAGTAACG |
| A369T-F | GGAAAATTCTTCCAT**ACC**GGGACAGATGAAGA |
| A369-R | ATGGAAGAATTTTCCGGAGAAACCTAAAT |
| Y384H-F | GATCTGCTTTTATCT**CAT**TCAAAGGAGTTCTGGTG |
| Y384-R | AGATAAAAGCAGATCATCACCAAGATCCT |
| S385A-F | CTGCTTTTATCTTAT**GCA**AAGGAGTTCTGGTG |
| S385-R | ATAAGATAAAAGCAGATCATCACCAAGAT |
| S385V-F | CTGCTTTTATCTTAT**GTT**AAGGAGTTCTGGTG |
| H417K-F | CAGATGTTGTTGAAC**AAA**AAGTTTGCAGAGGA |
| H417-R | GTTCAACAACATCTGCTCAGCAAGCACCG |
| N428D-F | CACGGCATTCCTACA**GAT**ATGGGATATGCCGT |
| N428-R | CACGGCATTCCTACA**CAT**ATGGGATATGCCGT |
| N428H-F | CACGGCATTCCTACA**CGT**ATGGGATATGCCGT |
| I443-R | TGGATAGACGCCGCTATGATGCGGTGCTACGG |
| I443V-F | AGCGGCGTCTATCCA**GTT**CACTTACAGTT |
| F473-R | ACGTGCAGGTTTCAAGTGCGGATATTCCT |
| F473Y-F | TTGAAACCTGCACGT**TAT**CGTCGTGGTTTCGT |
| V478I-F | TTCCGTCGTGGTTTC**ATT**CACTCGGGCATCTC |
| V478-R | GAAACCACGACGGAAACGTGCAGGTTTCAAGT |
| S480N-F | CGTGGTTTCGTCCAC**AAT**GGCATCTCTGTCTT |
| S480-R | GTGGACGAAACCACGACGGAAACGTGCAG |
| S483M-F | GTCCACTCGGGCATC**ATG**GTCTTACCACGCCAGAC |
| S483-R | GATGCCCGAGTGGACGAAACCACGACGGAAAC |
| G505/S506-R | ACCAGGGTACTCGTTATAGAAGATGGTGT |
| G505S-F | AACGAGTACCCTGGT**AGC**TCAAAAGAACTTGATAAATT |
| S506P-F | AACGAGTACCCTGGTGGC**CCG**AAAGAACTTGATAAATT |
| L512I-F | AAAGAACTTGATAAA**ATT**ATTTACGGTGGCGAATT |
| L512S-F | AAAGAACTTGATAAA**AGC**ATTTACGGTGGCGAATT |
| L512-R | TTTATCAAGTTCTTTTGAGCCACCAGGGT |
| S561K-F | ATGTGGACAAACCTT**AAA**CTGCAGACTTTACC |
| S561P-F | ATGTGGACAAACCTT**CCG**CTGCAGACTTTACC |
| S561R-F | ATGTGGACAAACCTT**CGT**CTGCAGACTTTACC |
| S561-R | AAGGTTTGTCCACATCTGGACAAATT |
| I577L-F | CAAAAGTACTTTCAG**CTG**TTCCCGGAAGAGCGTGACCC |
| I577-R | CTGAAAGTACTTTTGTGCAAGCTGCACGG |

Note: Homologous arm sequences are underlined, and mutations are shown in bold.

Table S3. Protein sequences of the key engineered proteins

| Name | Sequences |
| --- | --- |
| ND*^Dr^*-RL-ST | GVKRELEPAGAGGVVVPEEGTADWEDPRATPSPPSARVLPARTAKPADMSRTDPVVLVFVESLYSQLGQDIVAILESGRFRYQTEIAPGKGDMPTLTDKNRGRFTLVIYENILKYVNLDAWNRELLDKYCVEYGVGIIGFFKANENSLQSAQLKGFPLFLHSNLGLRDCSVNPKSPLLFITKAREVEHGPLPGDDWTVFQSNHTTYEPVLLARGRSTEASGAPGPLHAAVVQDLGLHDGIQRVLFGNNLNFWLHKLVLVDAVSFLTGKRLSLALERYILVDIDDIFVGKEGTRMKVSDVKALLETQNELRNHVPNFTFNLGFSGKFFHAGTDEEDLGDDLLLSYSKEFWWFPHMWSHMQPHLFHNQSVLAEQMLLNHKFAEEHGIPTNMGYAVAPHHSGVYPIHLQLYEAWKKVWGIKVTSTEEYPHLKPARFRRGFVHSGISVLPRQTCGLFTHTIFYNEYPGGSKELDKLIYGGELFLTVLLNPISIFMTHLSNYGNDRLGLYTFKNLVKFVQMWTNLSLQTLPPVQLAQKYFQIFPEERDPIWQDPCEDKRHKDIWSKEKTCDEAAAKRFPKLLIIGPQKTGTTALYLFLGMHPDLSSNYPSPETFEEIQFFNGHNYHKGIDWYMEFFPIPSNTTSDFYFEKSANYFDSEVAPRRAAALLPKAKVLTILINPADRAYSWYQHQRAHDDPVALKYTFHEVITAGSDAPSKLRALQNRCLVPGWYATHIERWLSAYHANQILVLDGNLLVTEPAKVMDMVQKFLGVTNTIDYHKTLAFDPKKGFWCQLLEGGKTKCLGKSKGRKYPPMDPDSRAFLKDYYRDHNIELSKLLYKMGQTLPTWLREDLQNTR |
| ND*^Dr^*^-WT^/ST | GVKRELEPAGAGGVVVPEEGTADWEDPRATPSPPSARVLPARTAKPADMSRTDPVVLVFVESLYSQLGQDIVAILESGRFRYQTEIAPGKGDMPTLTDKNRGRFTLVIYENILKYVNLDAWNRELLDKYCVEYGVGIIGFFKANENSLQSAQLKGFPLFLHSNLGLRDCSVNPKSPLL**F**ITKAREVEHGPLPGDDWTVFQSNHTTYEPVLLARGRSTEASGAPGPLHAAVVQDLGLHDGIQRVLFGNNLNFWLHKLVLVDAVSFLTGKRLSLALERYILVDIDDIFVGKEGTRMKVSDVKALLETQNELRNHVPNFTFNLGFSGKFFHAGTDEEDLGDDLLLSYSKEFWWFPHMWSHMQPHLFHNQSVLAEQMLLNHKFAEEHGIPTNMGYAVAPHHSGVYPIHLQLYEAWKKVWGIKVTSTEEYPHLKPARFRRGFVH**S**GISVLPRQTCGLFTHTIFYNEYPGGSKELDKLIYGGELFLTVLLNPISIFMTHLSNYGNDRLGLYTFKNLVKFVQMWTNLSLQTLPPVQLAQKYFQIFPEE**R**DPIWQDPCEDKRHKDIWSKEKTCDRFPKLLIIGPQKTGTTALYLFLGMHPDLSSNYPSPETFEEIQFFNGHNYHKGIDWYMEFFPIPSNTTSDFYFEKSANYFDSEVAPRRAAALLPKAKVLTILINPADRAYSWYQHQRAHDDPVALKYTFHEVITAGSDAPSKLRALQNRCLVPGWYATHIERWLSAYHANQILVLDGNLLVTEPAKVMDMVQKFLGVTNTIDYHKTLAFDPKKGFWCQLLEGGKTKCLGKSKGRKYPPMDPDSRAFLKDYYRDHNIELSKLLYKMGQTLPTWLREDLQNTR |
| ND*^Dr^*^-MEc^/ST | GVKRELEPAGAGGVVVPEEGTADWEDPRATPSPPSARVLPARTAKPADMSRTDPVVLVFVESLYSQLGQDIVAILESGRFRYQTEIAPGKGDMPTLTDKNRGRFTLVIYENILKYVNLDAWNRELLDKYCVEYGVGIIGFFKANENSLQSAQLKGFPLFLHSNLGLRDCSVNPKSPLL**H**ITKAREVEHGPLPGDDWTVFQSNHTTYEPVLLARGRSTEASGAPGPLHAAVVQDLGLHDGIQRVLFGNNLNFWLHKLVLVDAVSFLTGKRLSLALERYILVDIDDIFVGKEGTRMKVSDVKALLETQNELRNHVPNFTFNLGFSGKFFHAGTDEEDLGDDLLLSYSKEFWWFPHMWSHMQPHLFHNQSVLAEQMLLNHKFAEEHGIPTNMGYAVAPHHSGVYPIHLQLYEAWKKVWGIKVTSTEEYPHLKPARFRRGFVH**N**GISVLPRQTCGLFTHTIFYNEYPGGSKELDKLIYGGELFLTVLLNPISIFMTHLSNYGNDRLGLYTFKNLVKFVQMWTNLSLQTLPPVQLAQKYFQIFPEE**V**DPIWQDPCEDKRHKDIWSKEKTCDRFPKLLIIGPQKTGTTALYLFLGMHPDLSSNYPSPETFEEIQFFNGHNYHKGIDWYMEFFPIPSNTTSDFYFEKSANYFDSEVAPRRAAALLPKAKVLTILINPADRAYSWYQHQRAHDDPVALKYTFHEVITAGSDAPSKLRALQNRCLVPGWYATHIERWLSAYHANQILVLDGNLLVTEPAKVMDMVQKFLGVTNTIDYHKTLAFDPKKGFWCQLLEGGKTKCLGKSKGRKYPPMDPDSRAFLKDYYRDHNIELSKLLYKMGQTLPTWLREDLQNTR |

Note: Residues identical to the NST domain are underlined, and the mutation sites are indicated in bold.

Figure S1. Characterization of selected NDases towards chitin oligosaccharides. (A) UPLC chromatograms and (B) mass spectra of triacetylchitotriose and its deacetylated derivative generated by ND*^Sp^*. (C) UPLC chromatograms and (E) mass spectra of the deacetylated products of pentaacetylchitopentaose generated by ND*^Mr^*, ND*^Ar^*, ND*^Sl^*, ND*^Af^*, ND*^Hs^*, and ND*^Dr^*, and ND*^Pv^*. Deacetylated products were detected for ND*^Sp^*, ND*^Mr^*, ND*^Ar^*, ND*^Sl^*, and ND*^Af^*, whereas ND*^Hs^*, ND*^Dr^* and ND*^Pv^* showed no detectable activity toward pentaacetylchitopentaose.

Figure S2. Schematic representation of the deacetylase activity assay based on *N*-sulfotransferase-coupled detection system.

PAP, 3′-phosphoadenosine-5′-phosphate; AST-IV, aryl sulfotransferase IV; pNPS, *para*-nitrophenyl sulfate; pNP, *para*-nitrophenol.

Figure S3. Characterization of the deacetylases under different temperature (A), pH (B) and Co^2+^ concentration (C) conditions.

Relative activity was normalized to the maximum value of each dataset. Data are presented as mean ± SD (n = 3). The enzyme activity under reaction conditions used in this study (37 °C and pH 7.5) deviates by less than 20% from the maximum value under optimized conditions.

Figure S4. SDS-PAGE analysis of the artificial bifunctional enzymes

Expression of the artificial bifunctional enzymes in the intracellular supernatant (lanes 1, 3, 5, 7, 9 and 11) and inclusion bodies (lanes 2, 4, 6, 8, 10 and 12). Protein marker, lane M.

Figure S5. Mass spectra of the *N*-sulfated heparosan generated by the artificial NDSTs

Figure S6. Comparison of *N*-sulfation efficiency between the ND*^Dr^* cascade system and engineered bifunctional ND*^Dr^*-RL-ST.

Figure S7. Linker optimization of ND*^Mr^*-RL-ST

The rigid linkers (AEAAAKA)_2_ (RL2), (EAAAK)_2_ (RL3) and (PA)_3_P (RL4), as well as the **f**lexible **l**inkers such as G4S (FL1) and G6 (FL2), were employed between the ND*^Mr^* and NST domains to replace the original linker (RL) in the construction of the artificial ND*^Mr^*-RL-ST.

Figure S8. **Comparison of ND*^Mr^* and NST activities under cascade and one-pot reaction conditions.**

In the cascade reaction, NST sequentially catalyzed the products generated by ND*^Mr^*. In contrast, the one-pot reaction involved the simultaneous addition of ND*^Mr^* and NST to the reaction system for heparosan modification. Enzyme activity in the cascade reaction was set as 100% for reference, whereas the one-pot reaction exhibited a lower relative activity.

Figure S9. **Structural characterization of the interactions between distinct deacetylases and the *N*-sulfotransferase domain.** Interaction analysis between the NST domain (silver) and the ND*^Mr^* domain (purple) (A) or the ND*^Sl^* domain (cyan) (B). Yellow dashed lines represent the hydrogen bonds.

Figure S10. SDS-PAGE analysis of ND*^Dr^*/ST WT and its variants.

Figure S11. **Enzymatic characterization of ND*^Dr^*^-WT^/ST and ND*^Dr^*^-MEc^/ST.** (A) Optimal reaction temperatures and (B) optimal pH profiles.

Relative specific activities were normalized to the maximum specific activity of each enzyme (set as 100%).

Figure S12. **Apparent molecular weight distributions of heparosan and its modified products analyzed by GPC-HPLC.** GPC chromatogram of heparosan (A), enzymatically *N*-deacetylated heparosan generated by ND*^Dr^*^-MEc^/ST in the absence of PAPS (B), enzymatically *N*-sulfated heparosan produced by ND*^Dr^*^-MEc^/ST (C), and chemically *N*-deacetylated heparosan treated with NaOH (D).

Enzymatic reactions yielding *N*-deacetylated heparosan and *N*-sulfated heparosan reactions were terminated by heating at 100 °C for 10 min, followed by dialysis (10 kDa MWCO). Chemically *N*-deacetylated heparosan was prepared by NaOH treatment at 60 °C for 3 h,^[1]^ followed by dialysis (3.5 kDa MWCO), All samples were then followed by lyophilization and dissolution in H_2_O for GPC-HPLC analysis.

Figure S13. **^1^H NMR analysis of heparosan and its modified products.**

Figure S14. **^1^H-^13^C-heteronuclear single quantum coherence analysis of heparosan (A) and *N*-sulfated heparosan (B).**

Figure S15. **Validation of** ***N*-sulfated heparosan generated by ND*^Dr^*^-MEc^/ST.**

(A) Schematic illustration of the validation of enzymatically synthesized *N*-sulfated heparosan. (B) UPLC chromatograms of heparinase-depolymerized *N*-sulfated heparosan decasaccharides (10^AN^-5NS) generated by ND*^Dr^*^-MEc^/ST and *Homo sapiens* NDST expressed in *Komagataella phaffii* ^[2,3]^. (C) Mass spectrum of 10^AN^-5NS, with a characteristic ion at m/z 1052.7 corresponding to [M–3H+Na]^2−^. (D) Schematic illustration of the downstream enzymatic modification of enzymatically synthesized *N*-sulfated heparosan. (E) UPLC-MS analysis of *N*- and 2-*O*-sulfated heparosan. *N*-sulfated heparosan was further treated with C5-epimerase (C5-epi) and 2-*O*-sulfotransferase (2-*O*-ST)^[4]^ to verify its suitability for downstream enzymatic modification. The characteristic disaccharide ΔUA2S-GlcNS was detected with an [M–H]^−^ ion at m/z 496.02.

**Reference:**

[1] Z. Wang, J. Li, S. Cheong, et al., Response surface optimization of the heparosan *N*-deacetylation in producing bioengineered heparin. *J. Biotechnol.* 2011, *156* (3), 188–196.

[2] Y. Zhang, Y. Wang, Z. Zhou, et al., Synthesis of bioengineered heparin by recombinant yeast Pichia pastoris. *Green Chem.* 2022, 24 (8), 3180–3192.

[3] X. Xi, W. Zhang, L. Hu, et al., Enzymatic construction of a library of even- and odd-numbered heparosan oligosaccharides and their *N*-sulfonated derivatives. *Int. J. Biol. Macromol*. 2024, 264, 130501.

[4] W. Chen, D. Wang, X. Xi, et al., Efficient expression of an engineered heparan sulfate 2-*O*-sulfotransferase with improved catalytic properties. *J. Agric. Food Chem.* 2025, *73* (21), 12856–12866.
