# Supplementary material for: Design and Engineering of an Artificial Bifunctional N‐Deacetylase/N‐Sulfotransferase for the Biosynthesis of N‐Sulfated Heparosan
Source: Adv Sci (Weinh). 2026 Jul 10:e76499. Online ahead of print. doi: 10.1002/advs.76499 (PMC13353177; doi:10.1002/advs.76499)
Supplement: Supplementary file 1 — Supporting File: advs76499‐sup‐0001‐SuppMat.docx. [file ADVS-9999-e76499-s001.docx]

Supporting Information

Design and Engineering of an Artificial Bifunctional *N*-Deacetylase/*N*-Sulfotransferase for the Biosynthesis of *N*-Sulfated Heparosan

*Xintong Xi^1,2,3^*, *Ruirui Xu^1,3,4^*, *Daoan Wang^1,3,4^*, *Guobin Yin^1,3,4^*, *Xinjing Wang^1,3,4^*, *Haiqin Chen^2^*, *Guocheng Du^1,3,4^*, *Jian Chen^1,3,4^*, *Zhen Kang^1,3,4*^*

^1^The Key Laboratory of Carbohydrate Chemistry and Biotechnology, Ministry of Education, Jiangnan University, Wuxi, 214122, China

^2^School of Food Science and Technology, Jiangnan University, Wuxi, 214122, China ^3^The Science Center for Future Foods, Jiangnan University, Wuxi, 214122, China

^4^Jiangsu Province Basic Research Center for Synthetic Biology, Jiangnan University, Wuxi, 214122, China

*Corresponding author

E-mail for Zhen Kang: [zkang@jiangnan.edu.cn](mailto:zkang@jiangnan.edu.cn)

Phone: +86-510-85918307, Fax: +86-510-85918309 (Z. Kang)

Table S1. Strains and plasmids used in this study

| Strains or plasmids | Characteristics | Source |
| --- | --- | --- |
| *E. coli* strains |  |  |
| *E. coli* JM109 | Cloning host | Invitrogen |
| *E. coli* Rosetta (DE3) | Expression host, Cm^R^ | Invitrogen |
| Plasmids |  |  |
| pET-32a (+) | Expression vector, Amp^R^ | Novagen |
| pET32a-ND*^Sp^* | pET-32a (+) carrying *N*-deacetylase gene of *Streptococcus pneumoniae* (UniProtKB—A0A0H2ZN69), Amp^R^ | This work |
| pET32a-ND*^Mr^* | pET-32a (+) carrying MBP gene and *N*-deacetylase gene of *Mucor rouxii* (UniProtKB—P50325) with flexible linker, Amp^R^ | This work |
| pET32a-ND*^Ar^* | pET-32a (+) carrying MBP gene and *N*-deacetylase gene of *Arthrobacter sp* (UniParc—UPI000B8BB35C) with flexible linker, Amp^R^ | This work |
| pET32a-ND*^Sl^* | pET-32a (+) carrying MBP gene and *N*-deacetylase gene of *Streptomyces lividans* (UniProtKB—A0ABM5R7G1) with flexible linker, Amp^R^ | This work |
| pET32a-ND*^Af^* | pET-32a (+) carrying MBP gene and *N*-deacetylase gene of *Aspergillus fumigatus* (UniProtKB—Q4WX15) with flexible linker, Amp^R^ | This work |
| pET32a-ND*^Hs^* | pET-32a (+) carrying MBP gene and *N*-deacetylase gene of *Homo sapiens* (UniProtKB—P52848) with flexible linker, Amp^R^ | This work |
| pET32a-ND*^Dr^* | pET-32a (+) carrying MBP gene and *N*-deacetylase gene of *Danio rerio* (UniProtKB—A0A0R4IR84) with flexible linke, Amp^R^ | This work |
| pET32a-ND*^Pv^* | pET-32a (+) carrying MBP gene and *N*-deacetylase gene of *Penaeus vannamei* (UniParc—UPI000F662199) with flexible linker, Amp^R^ | This work |
| pET32a-ND*^Mr^*-RL-ST | pET32a-ND*^Mr^* carrying NST gene with rigid linker, Amp^R^ | This work |
| pET32a-ND*^Ar^*- RL-ST | pET32a-ND*^Ar^* carrying NST gene with rigid linker, Amp^R^ | This work |
| pET32a-ND*^Sl^*- RL-ST | pET32a-ND*^Sl^* carrying NST gene with rigid linker, Amp^R^ | This work |
| pET32a-ND*^Af^*- RL-ST | pET32a-ND*^Af^* carrying NST gene with rigid linker, Amp^R^ | This work |
| pET32a-ND*^Dr^*-RL-ST | pET32a-ND*^Dr^* carrying NST gene with rigid linker, Amp^R^ | This work |
| pET32a-ND*^Pv^*-RL-ST | pET32a-ND*^Pv^* carrying NST gene with rigid linker, Amp^R^ | This work |

Table S2. Primers used in this study

| Name | Sequences (5' - 3') |
| --- | --- |
| vector-F | TAAAAGCTTGCGGCCGCACTCGAGC |
| vector-R | GGATCCGATATCAGCCATGGCCTTGTCGT |
| MBP-F | ATGGCTGATATCGGATCCATGAAAATCGAAGAAGGTAAACTGGT |
| MBP-R | GCTGCCGCCGCCGCCAGTCTGCGCGTCTTTCAGG |
| ND*^Sp^*-F | GCCATGGCTGATATCGGATCCATGAGCAATTGCATCATCGAAATCAT |
| ND*^Sp^*-R | GTGCGGCCGCAAGCTTTTATTCATCGCGGCTGTAGTACAGT |
| ND*^Mr^*-F | GGCGGCGGCGGCAGCGATACCAGCGCCAATTACTGGCAGAG |
| ND*^Mr^*-R | GTGCGGCCGCAAGCTTTTAAACATTGGTCCATTCGTAATCTTCGAAG |
| ND*^Ar^*-F | CAGACTGGCGGCGGCGGCAGCGCTGGACAACCTGAACCAGTAGC |
| ND*^Ar^*-R | AGTGCGGCCGCAAGCTTTTACGGGTTCGTCTTAAAACGATGGT |
| ND*^Sl^*-F | ACTGGCGGCGGCGGCAGCGCTGCATGTAATGGATATGTAGGGCT |
| ND*^Sl^*-R | AGTGCGGCCGCAAGCTTTTAGCCGGCGGTACAGCTGACAGATG |
| ND*^Af^*-F | ACTGGCGGCGGCGGCAGCCACGGCAGCGTTCTGAGCAATAT |
| ND*^Af^*-R | AGTGCGGCCGCAAGCTTTTATTCCAGGGCGATCGGGGTGCTCA |
| ND*^Dr^* -F | GGCGGCGGCGGCAGCGGCGTAAAGCGCGAACTTGAACC |
| ND*^Dr^* -R | GTGCGGCCGCAAGCTTTTATTTAGGAAAACGATCACACGTCTTTTCCT |
| ND*^Hs^* -F | GGCGGCGGCGGCAGCGGATGGAAAAGGGGGCTAGAACC |
| ND*^Hs^* -R | CTCGAGTGCGGCCGCAAGCTTTTATTTTGGGAAGCGATCACAGGTCTTCT |
| ND*^Dr^* -ST-R | ATCACACGTCTTTTCCTTCGACCAGATATCCT |
| ND*^Dr^* -ST-F | TCGAAGGAAAAGACGTGTGATAGATTCCCTAAGTTGTTGATCAT |
| ND*^Mr^*-RL-ST-F | GAATGGACCAATGTTGAAGCGGCGGCGAAAAGATTCCCTAAGTTGTTGATCAT |
| ND*^Ar^* -RL-ST-F | TTTAAGACGAACCCGGAAGCGGCGGCGAAAAGATTCCCTAAGTTGTTGATCAT |
| ND*^Sl^* -RL-ST-F | AGCTGTACCGCCGGCGAAGCGGCGGCGAAAAGATTCCCTAAGTTGTTGATCAT |
| ND*^Af^* -RL-ST-F | CCGATCGCCCTGGAAGAAGCGGCGGCGAAAAGATTCCCTAAGTTGTTGATCAT |
| ND*^Dr^* -RL-ST-F | GAATGGACCAATGTTGAAGCGGCGGCGAAAAGATTCCCTAAGTTGTTGATCAT |
| ND*^Pv^* -RL-ST-F | ATCTGGTCCAAGAACGAAGCGGCGGCGAAAAGATTCCCTAAGTTGTTGATCAT |
| G6-ST-F | GAATGGACCAATGTTGGTGGCGGTGGCGGCGGTAGATTCCCTAAGTTGTTGATCAT |
| G4S-ST-F | GAATGGACCAATGTTGGCGGTGGTGGTAGCAGATTCCCTAAGTTGTTGATCAT |
| ST-R | TGCGGCCGCAAGCTTTTATCTAGTATTTTGCAAATCCTCTCTCAACC |
| Nolinker-MBP-R | AGTCTGCGCGTCTTTCAGGGCTTCAT |
| MBP-ND*^Dr^* -F | GGCGTAAAGCGCGAACTTGAACCAGC |
| MBP-G4S2-R | AGAACCACCACCACCTGAACCACCGCCACCAGTCTGCGCGTCTTTCAGGG |
| MBP-EAAAK-R | TTTGGCGGCCGCTTCAGTCTGCGCGTCTTTCAGGG |
| MBP-G6-R | GCCTCCACCGCCGCCGCCAGTCTGCGCGTCTTTCAGGG |
| MBP-PT4-R | GGTCGGGGTCGGGGTCGGGGTCGGAGTCTGCGCGTCTTTCAGGG |
| MBP-PT2-R | GGTCGGGGTCGGAGTCTGCGCGTCTTTCAGGG |
| D110E-F | AGTCAGTTGGGCCAG**GAA**ATTGTCGCCATCTT |
| D110/I111-R | CTGGCCCAACTGACTGTACAGTGATT |
| I111V-F | AGTCAGTTGGGCCAGGAT**GTT**GTCGCCATCTT |
| G118S-F | GCCATCTTGGAGTCC**AGC**CGCTTCCGCTATCAAAC |
| G118-R | GGACTCCAAGATGGCGACAATATCCTGGC |
| Q189L-F | AATGAAAATTCTTTG**CTG**TCTGCTCAGTTGAAAGG |
| Q189-R | CAAAGAATTTTCATTTGCTTTGAAGAAAC |
| F219H-F | AAATCGCCACTTTTA**CAT**ATCACCAAGGCGCG |
| F219-R | TAAAAGTGGCGATTTGGGGTTGACGGAGC |
| F219Y-F | AAATCGCCACTTTTA**TAT**ATCACCAAGGCGCG |
| K222R-F | CTTTTATTTATCACC**CGT**GCGCGCGAAGTAGAGCATGG |
| K222-R224-R | GGTGATAAATAAAAGTGGCGATTTGGGGT |
| A223P-F | CTTTTATTTATCACCAAG**CCG**CGCGAAGTAGAGCATGG |
| A223S-F | CTTTTATTTATCACCAAG**AGC**CGCGAAGTAGAGCATGG |
| R224F-F | CTTTTATTTATCACCAAGGCG**GGT**GAAGTAGAGCATGG |
| R224S-F | CTTTTATTTATCACCAAGGCG**AGC**GAAGTAGAGCATGG |
| E227/H228-R | TACTTCGCGCGCCTTGGTGATAAATAAAAGTG |
| E227L-F | AAGGCGCGCGAAGTA**CTG**CATGGCCCTCTGCC |
| H228K-F | AAGGCGCGCGAAGTAGAG**AAA**GGCCCTCTGCCTGG |
| H228P-F | AAGGCGCGCGAAGTAGAG**CCG**GGCCCTCTGCCTGG |
| P230-R | GCCATGCTCTACTTCGCGCGCCTTGG |
| P230V-F | GAAGTAGAGCATGGC**GTT**CTGCCTGGCGATGATTG |
| D234E-F | GGCCCTCTGCCTGGC**GAA**GATTGGACCGT |
| D234-R | GCCAGGCAGAGGGCCATGCTCTACTT |
| T244S-F | TTTCAATCCAATCAT**AGC**ACCTATGAGCCTGT |
| T244-R | ATGATTGGATTGAAAGACGGTCCAATCATCGC |
| G254T-F | CCTGTGCTGCTGGCGCGC**ACC**CGTTCCACTGAAGC |
| G254L-F | CCTGTGCTGCTGGCGCGC**CTG**CGTTCCACTGAAGC |
| R255K-F | CCTGTGCTGCTGGCGCGCGGC**AAA**TCCACTGAAGC |
| R255Q-F | CCTGTGCTGCTGGCGCGCGGC**CAG**TCCACTGAAGC |
| G254/R255-R | CGCCAGCAGCACAGGCTCATAGGTGGTAT |
| A259S-F | GGCCGTTCCACTGAA**AGC**AGTGGCGCCCCAGGCCC |
| A259/S260-R | TTCAGTGGAACGGCCGCGCGCCAGCAGCACAG |
| S260I-F | GGCCGTTCCACTGAAGCA**ATT**GGCGCCCCAGGCCC |
| S260V-F | GGCCGTTCCACTGAAGCA**GTT**GGCGCCCCAGGCCC |
| A269S-F | GGCCCATTACATGCC**AGC**GTTGTTCAGGACTT |
| A269T-F | GGCCCATTACATGCC**ACC**GTTGTTCAGGACTT |
| A269-R | GGCATGTAATGGGCCTGGGGCGCCACTTG |
| L298F-F | CTTCACAAGTTAGTG**TTT**GTTGACGCGGTGTC |
| L298-R | CACTAACTTGTGAAGCCAAAAGTTAAGAT |
| A313G-F | AAGCGCTTATCGTTA**GGT**TTGGAACGTTATAT |
| A313P-F | AAGCGCTTATCGTTA**CCG**TTGGAACGTTATAT |
| A313S-F | AAGCGCTTATCGTTA**AGC**TTGGAACGTTATAT |
| A313-R | TAACGATAAGCGCTTGCCTGTAAGGAACG |
| N351T-F | CAAAATGAATTGCGT**ACC**CACGTACCGAATTT |
| N351-R | ACGCAATTCATTTTGTGTTTCAAGTAACG |
| A369T-F | GGAAAATTCTTCCAT**ACC**GGGACAGATGAAGA |
| A369-R | ATGGAAGAATTTTCCGGAGAAACCTAAAT |
| Y384H-F | GATCTGCTTTTATCT**CAT**TCAAAGGAGTTCTGGTG |
| Y384-R | AGATAAAAGCAGATCATCACCAAGATCCT |
| S385A-F | CTGCTTTTATCTTAT**GCA**AAGGAGTTCTGGTG |
| S385-R | ATAAGATAAAAGCAGATCATCACCAAGAT |
| S385V-F | CTGCTTTTATCTTAT**GTT**AAGGAGTTCTGGTG |
| H417K-F | CAGATGTTGTTGAAC**AAA**AAGTTTGCAGAGGA |
| H417-R | GTTCAACAACATCTGCTCAGCAAGCACCG |
| N428D-F | CACGGCATTCCTACA**GAT**ATGGGATATGCCGT |
| N428-R | CACGGCATTCCTACA**CAT**ATGGGATATGCCGT |
| N428H-F | CACGGCATTCCTACA**CGT**ATGGGATATGCCGT |
| I443-R | TGGATAGACGCCGCTATGATGCGGTGCTACGG |
| I443V-F | AGCGGCGTCTATCCA**GTT**CACTTACAGTT |
| F473-R | ACGTGCAGGTTTCAAGTGCGGATATTCCT |
| F473Y-F | TTGAAACCTGCACGT**TAT**CGTCGTGGTTTCGT |
| V478I-F | TTCCGTCGTGGTTTC**ATT**CACTCGGGCATCTC |
| V478-R | GAAACCACGACGGAAACGTGCAGGTTTCAAGT |
| S480N-F | CGTGGTTTCGTCCAC**AAT**GGCATCTCTGTCTT |
| S480-R | GTGGACGAAACCACGACGGAAACGTGCAG |
| S483M-F | GTCCACTCGGGCATC**ATG**GTCTTACCACGCCAGAC |
| S483-R | GATGCCCGAGTGGACGAAACCACGACGGAAAC |
| G505/S506-R | ACCAGGGTACTCGTTATAGAAGATGGTGT |
| G505S-F | AACGAGTACCCTGGT**AGC**TCAAAAGAACTTGATAAATT |
| S506P-F | AACGAGTACCCTGGTGGC**CCG**AAAGAACTTGATAAATT |
| L512I-F | AAAGAACTTGATAAA**ATT**ATTTACGGTGGCGAATT |
| L512S-F | AAAGAACTTGATAAA**AGC**ATTTACGGTGGCGAATT |
| L512-R | TTTATCAAGTTCTTTTGAGCCACCAGGGT |
| S561K-F | ATGTGGACAAACCTT**AAA**CTGCAGACTTTACC |
| S561P-F | ATGTGGACAAACCTT**CCG**CTGCAGACTTTACC |
| S561R-F | ATGTGGACAAACCTT**CGT**CTGCAGACTTTACC |
| S561-R | AAGGTTTGTCCACATCTGGACAAATT |
| I577L-F | CAAAAGTACTTTCAG**CTG**TTCCCGGAAGAGCGTGACCC |
| I577-R | CTGAAAGTACTTTTGTGCAAGCTGCACGG |

Note: Homologous arm sequences are underlined, and mutations are shown in bold.

Table S3. Protein sequences of the key engineered proteins

| Name | Sequences |
| --- | --- |
| ND*^Dr^*-RL-ST | GVKRELEPAGAGGVVVPEEGTADWEDPRATPSPPSARVLPARTAKPADMSRTDPVVLVFVESLYSQLGQDIVAILESGRFRYQTEIAPGKGDMPTLTDKNRGRFTLVIYENILKYVNLDAWNRELLDKYCVEYGVGIIGFFKANENSLQSAQLKGFPLFLHSNLGLRDCSVNPKSPLLFITKAREVEHGPLPGDDWTVFQSNHTTYEPVLLARGRSTEASGAPGPLHAAVVQDLGLHDGIQRVLFGNNLNFWLHKLVLVDAVSFLTGKRLSLALERYILVDIDDIFVGKEGTRMKVSDVKALLETQNELRNHVPNFTFNLGFSGKFFHAGTDEEDLGDDLLLSYSKEFWWFPHMWSHMQPHLFHNQSVLAEQMLLNHKFAEEHGIPTNMGYAVAPHHSGVYPIHLQLYEAWKKVWGIKVTSTEEYPHLKPARFRRGFVHSGISVLPRQTCGLFTHTIFYNEYPGGSKELDKLIYGGELFLTVLLNPISIFMTHLSNYGNDRLGLYTFKNLVKFVQMWTNLSLQTLPPVQLAQKYFQIFPEERDPIWQDPCEDKRHKDIWSKEKTCDEAAAKRFPKLLIIGPQKTGTTALYLFLGMHPDLSSNYPSPETFEEIQFFNGHNYHKGIDWYMEFFPIPSNTTSDFYFEKSANYFDSEVAPRRAAALLPKAKVLTILINPADRAYSWYQHQRAHDDPVALKYTFHEVITAGSDAPSKLRALQNRCLVPGWYATHIERWLSAYHANQILVLDGNLLVTEPAKVMDMVQKFLGVTNTIDYHKTLAFDPKKGFWCQLLEGGKTKCLGKSKGRKYPPMDPDSRAFLKDYYRDHNIELSKLLYKMGQTLPTWLREDLQNTR |
| ND*^Dr^*^-WT^/ST | GVKRELEPAGAGGVVVPEEGTADWEDPRATPSPPSARVLPARTAKPADMSRTDPVVLVFVESLYSQLGQDIVAILESGRFRYQTEIAPGKGDMPTLTDKNRGRFTLVIYENILKYVNLDAWNRELLDKYCVEYGVGIIGFFKANENSLQSAQLKGFPLFLHSNLGLRDCSVNPKSPLL**F**ITKAREVEHGPLPGDDWTVFQSNHTTYEPVLLARGRSTEASGAPGPLHAAVVQDLGLHDGIQRVLFGNNLNFWLHKLVLVDAVSFLTGKRLSLALERYILVDIDDIFVGKEGTRMKVSDVKALLETQNELRNHVPNFTFNLGFSGKFFHAGTDEEDLGDDLLLSYSKEFWWFPHMWSHMQPHLFHNQSVLAEQMLLNHKFAEEHGIPTNMGYAVAPHHSGVYPIHLQLYEAWKKVWGIKVTSTEEYPHLKPARFRRGFVH**S**GISVLPRQTCGLFTHTIFYNEYPGGSKELDKLIYGGELFLTVLLNPISIFMTHLSNYGNDRLGLYTFKNLVKFVQMWTNLSLQTLPPVQLAQKYFQIFPEE**R**DPIWQDPCEDKRHKDIWSKEKTCDRFPKLLIIGPQKTGTTALYLFLGMHPDLSSNYPSPETFEEIQFFNGHNYHKGIDWYMEFFPIPSNTTSDFYFEKSANYFDSEVAPRRAAALLPKAKVLTILINPADRAYSWYQHQRAHDDPVALKYTFHEVITAGSDAPSKLRALQNRCLVPGWYATHIERWLSAYHANQILVLDGNLLVTEPAKVMDMVQKFLGVTNTIDYHKTLAFDPKKGFWCQLLEGGKTKCLGKSKGRKYPPMDPDSRAFLKDYYRDHNIELSKLLYKMGQTLPTWLREDLQNTR |
| ND*^Dr^*^-MEc^/ST | GVKRELEPAGAGGVVVPEEGTADWEDPRATPSPPSARVLPARTAKPADMSRTDPVVLVFVESLYSQLGQDIVAILESGRFRYQTEIAPGKGDMPTLTDKNRGRFTLVIYENILKYVNLDAWNRELLDKYCVEYGVGIIGFFKANENSLQSAQLKGFPLFLHSNLGLRDCSVNPKSPLL**H**ITKAREVEHGPLPGDDWTVFQSNHTTYEPVLLARGRSTEASGAPGPLHAAVVQDLGLHDGIQRVLFGNNLNFWLHKLVLVDAVSFLTGKRLSLALERYILVDIDDIFVGKEGTRMKVSDVKALLETQNELRNHVPNFTFNLGFSGKFFHAGTDEEDLGDDLLLSYSKEFWWFPHMWSHMQPHLFHNQSVLAEQMLLNHKFAEEHGIPTNMGYAVAPHHSGVYPIHLQLYEAWKKVWGIKVTSTEEYPHLKPARFRRGFVH**N**GISVLPRQTCGLFTHTIFYNEYPGGSKELDKLIYGGELFLTVLLNPISIFMTHLSNYGNDRLGLYTFKNLVKFVQMWTNLSLQTLPPVQLAQKYFQIFPEE**V**DPIWQDPCEDKRHKDIWSKEKTCDRFPKLLIIGPQKTGTTALYLFLGMHPDLSSNYPSPETFEEIQFFNGHNYHKGIDWYMEFFPIPSNTTSDFYFEKSANYFDSEVAPRRAAALLPKAKVLTILINPADRAYSWYQHQRAHDDPVALKYTFHEVITAGSDAPSKLRALQNRCLVPGWYATHIERWLSAYHANQILVLDGNLLVTEPAKVMDMVQKFLGVTNTIDYHKTLAFDPKKGFWCQLLEGGKTKCLGKSKGRKYPPMDPDSRAFLKDYYRDHNIELSKLLYKMGQTLPTWLREDLQNTR |

Note: Residues identical to the NST domain are underlined, and the mutation sites are indicated in bold.

**
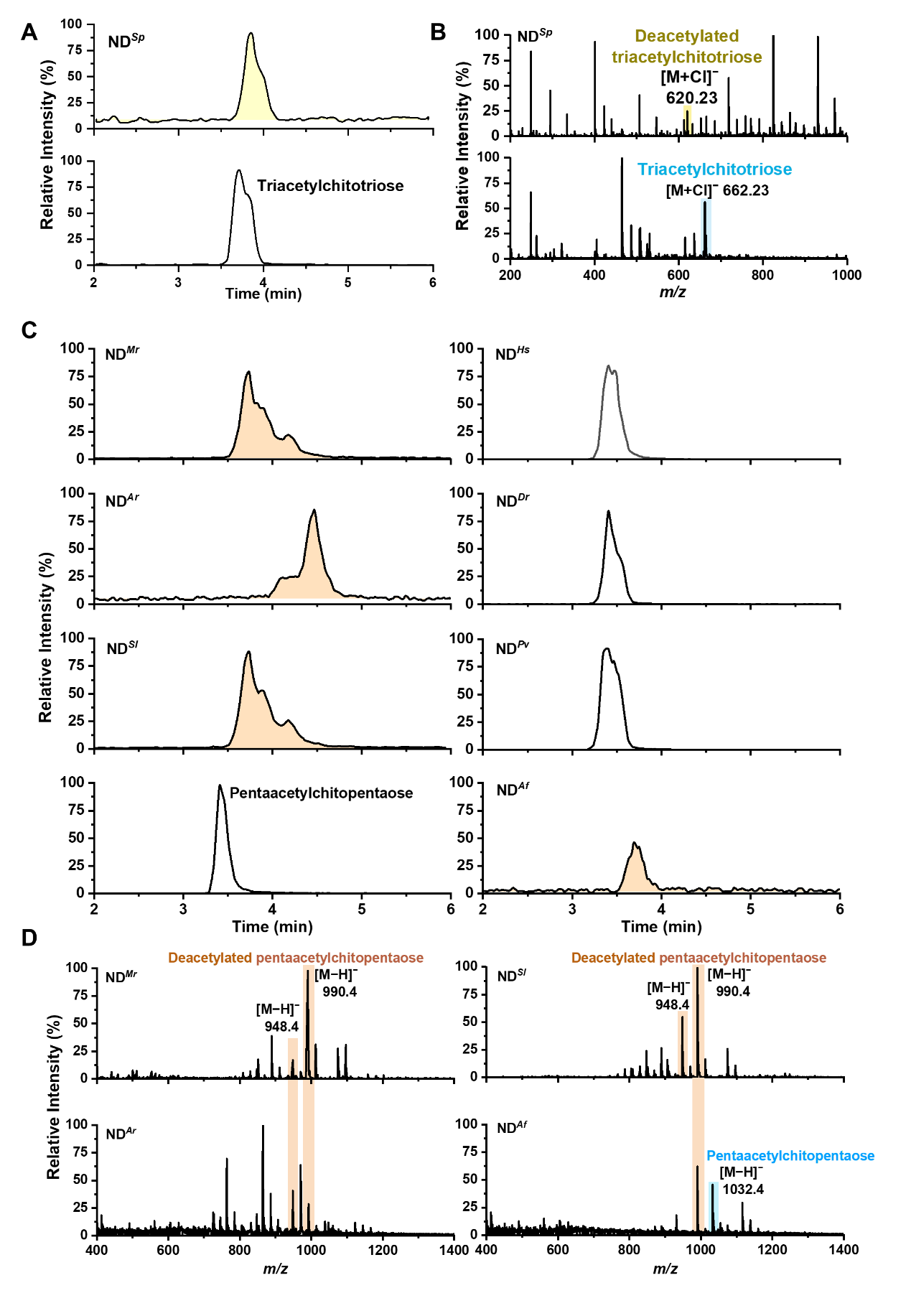
**

Figure S1. Characterization of selected NDases towards chitin oligosaccharides. (A) UPLC chromatograms and (B) mass spectra of triacetylchitotriose and its deacetylated derivative generated by ND*^Sp^*. (C) UPLC chromatograms and (E) mass spectra of the deacetylated products of pentaacetylchitopentaose generated by ND*^Mr^*, ND*^Ar^*, ND*^Sl^*, ND*^Af^*, ND*^Hs^*, and ND*^Dr^*, and ND*^Pv^*. Deacetylated products were detected for ND*^Sp^*, ND*^Mr^*, ND*^Ar^*, ND*^Sl^*, and ND*^Af^*, whereas ND*^Hs^*, ND*^Dr^* and ND*^Pv^* showed no detectable activity toward pentaacetylchitopentaose.


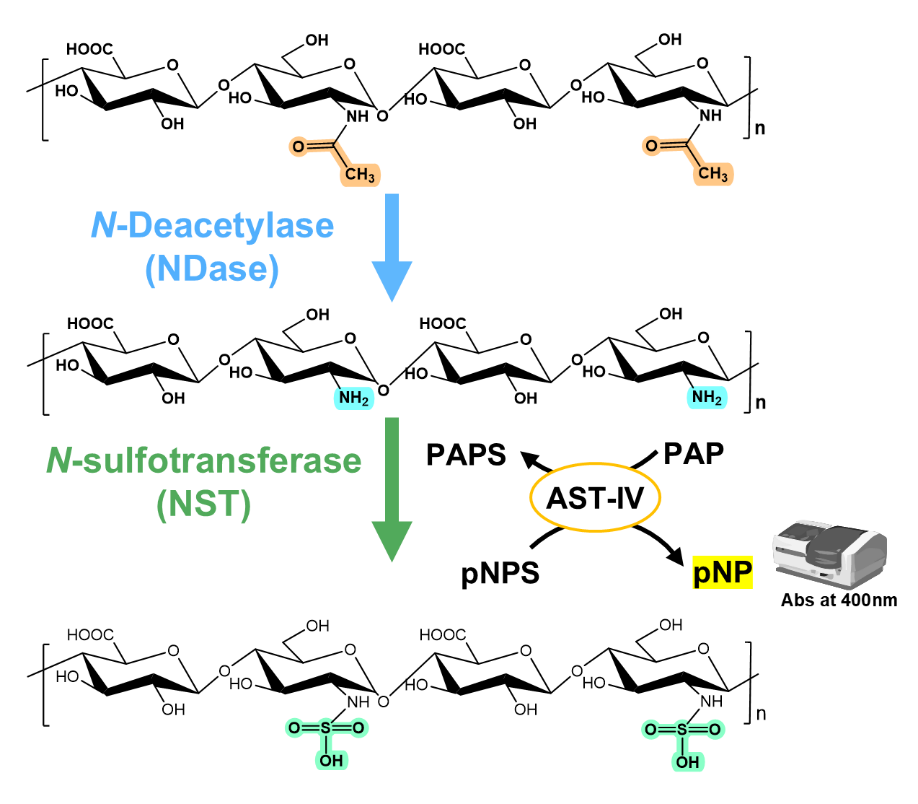


Figure S2. Schematic representation of the deacetylase activity assay based on *N*-sulfotransferase-coupled detection system.

PAP, 3′-phosphoadenosine-5′-phosphate; AST-IV, aryl sulfotransferase IV; pNPS, *para*-nitrophenyl sulfate; pNP, *para*-nitrophenol.


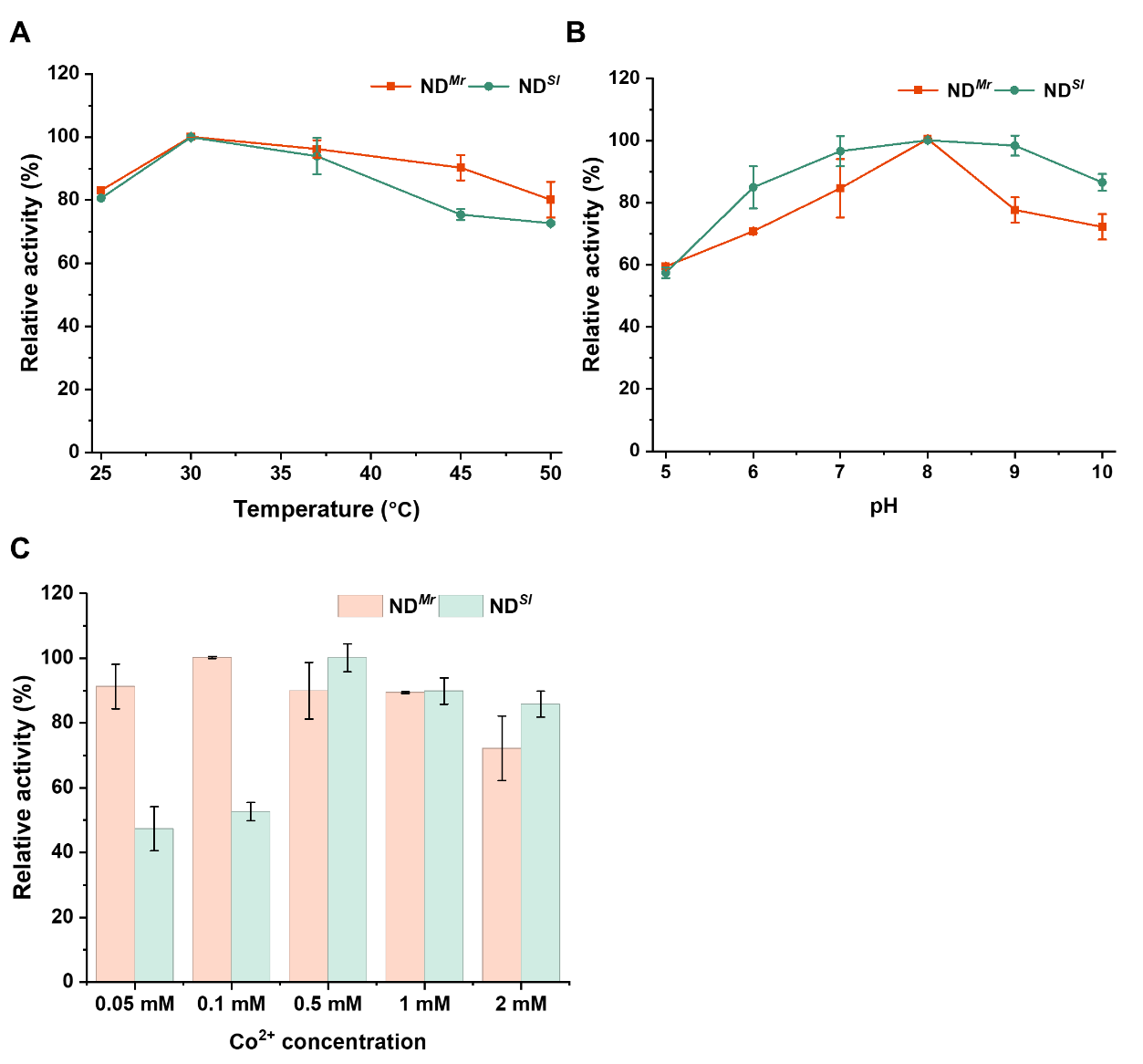
Figure S3. Characterization of the deacetylases under different temperature (A), pH (B) and Co^2+^ concentration (C) conditions.

Relative activity was normalized to the maximum value of each dataset. Data are presented as mean ± SD (n = 3). The enzyme activity under reaction conditions used in this study (37 °C and pH 7.5) deviates by less than 20% from the maximum value under optimized conditions.

**
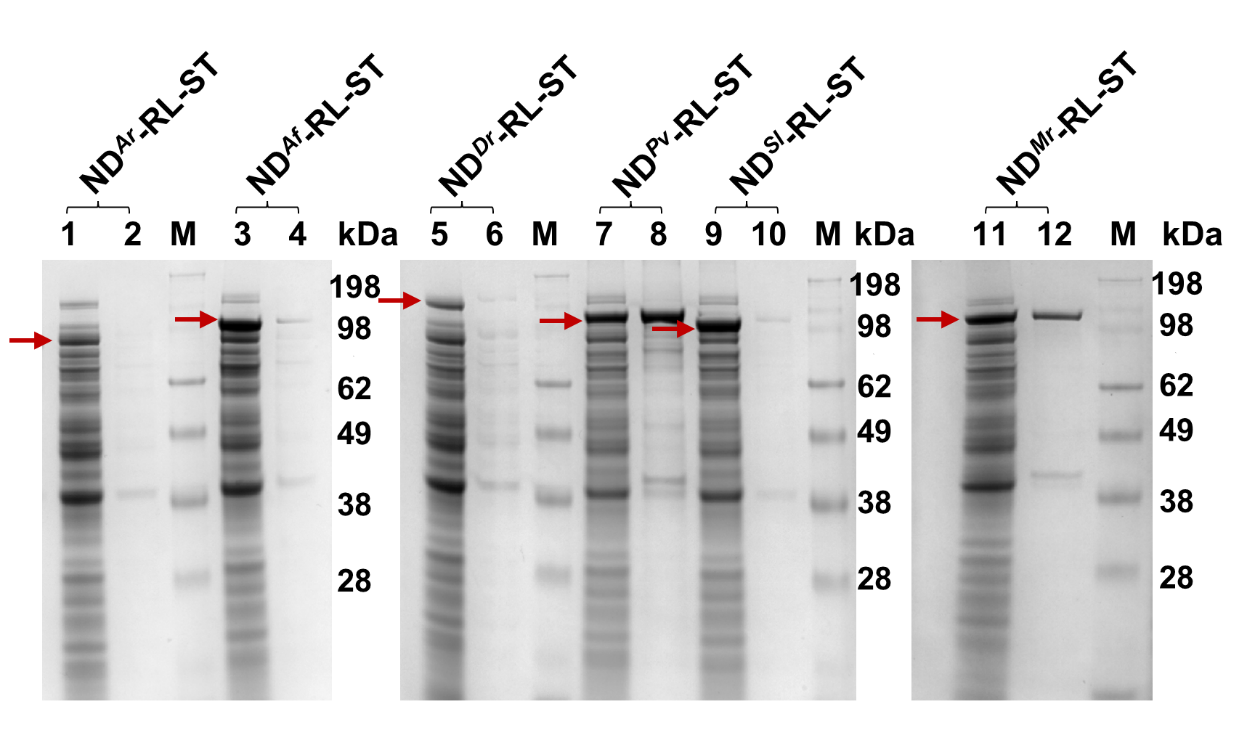
**

Figure S4. SDS-PAGE analysis of the artificial bifunctional enzymes

Expression of the artificial bifunctional enzymes in the intracellular supernatant (lanes 1, 3, 5, 7, 9 and 11) and inclusion bodies (lanes 2, 4, 6, 8, 10 and 12). Protein marker, lane M.


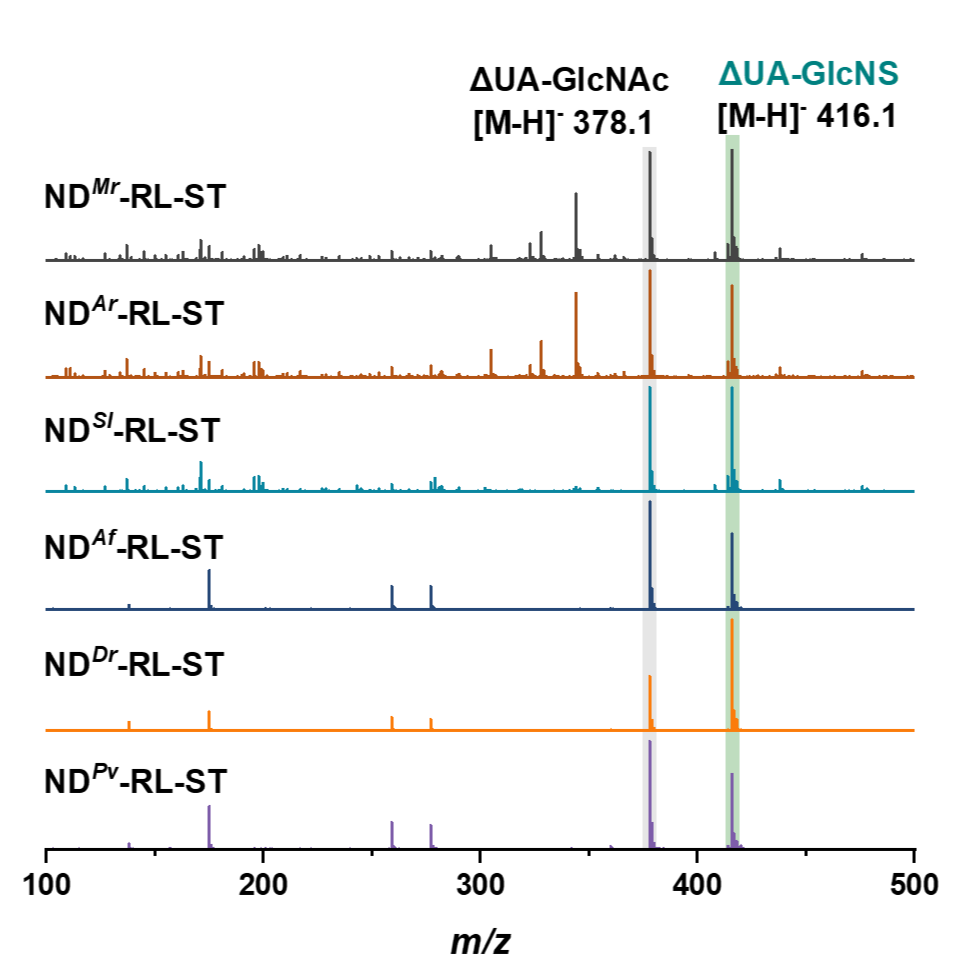


Figure S5. Mass spectra of the *N*-sulfated heparosan generated by the artificial NDSTs


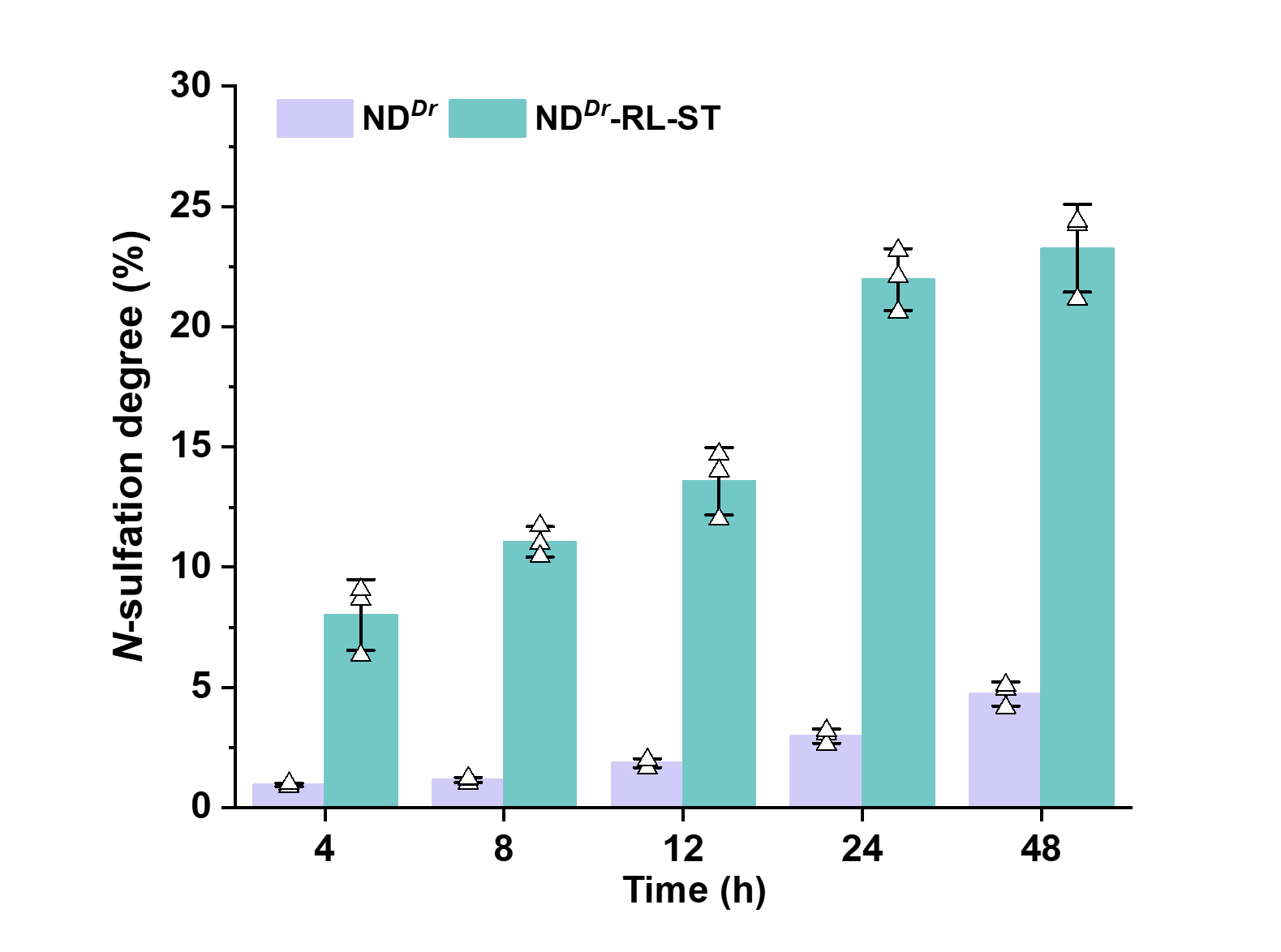


Figure S6. Comparison of *N*-sulfation efficiency between the ND*^Dr^* cascade system and engineered bifunctional ND*^Dr^*-RL-ST.


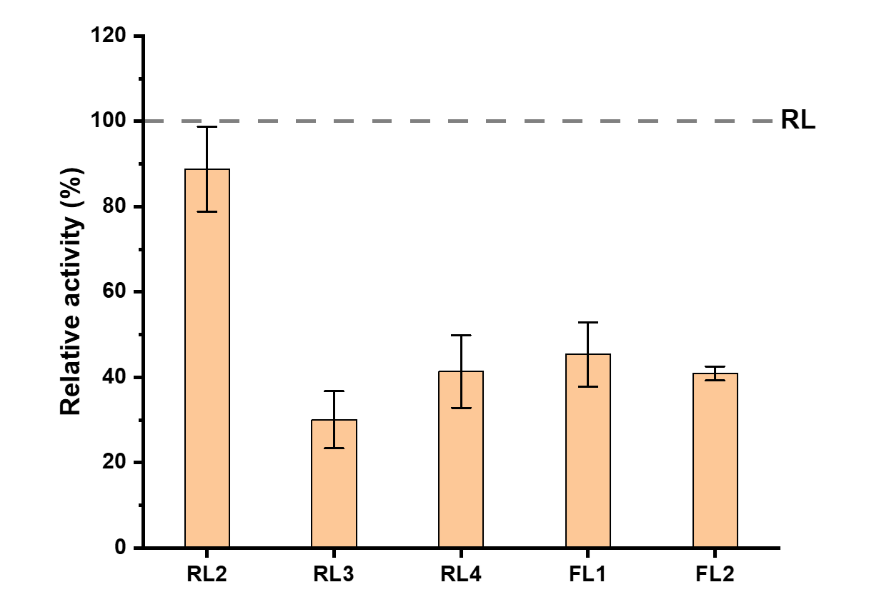


Figure S7. Linker optimization of ND*^Mr^*-RL-ST

The rigid linkers (AEAAAKA)_2_ (RL2), (EAAAK)_2_ (RL3) and (PA)_3_P (RL4), as well as the **f**lexible **l**inkers such as G4S (FL1) and G6 (FL2), were employed between the ND*^Mr^* and NST domains to replace the original linker (RL) in the construction of the artificial ND*^Mr^*-RL-ST.


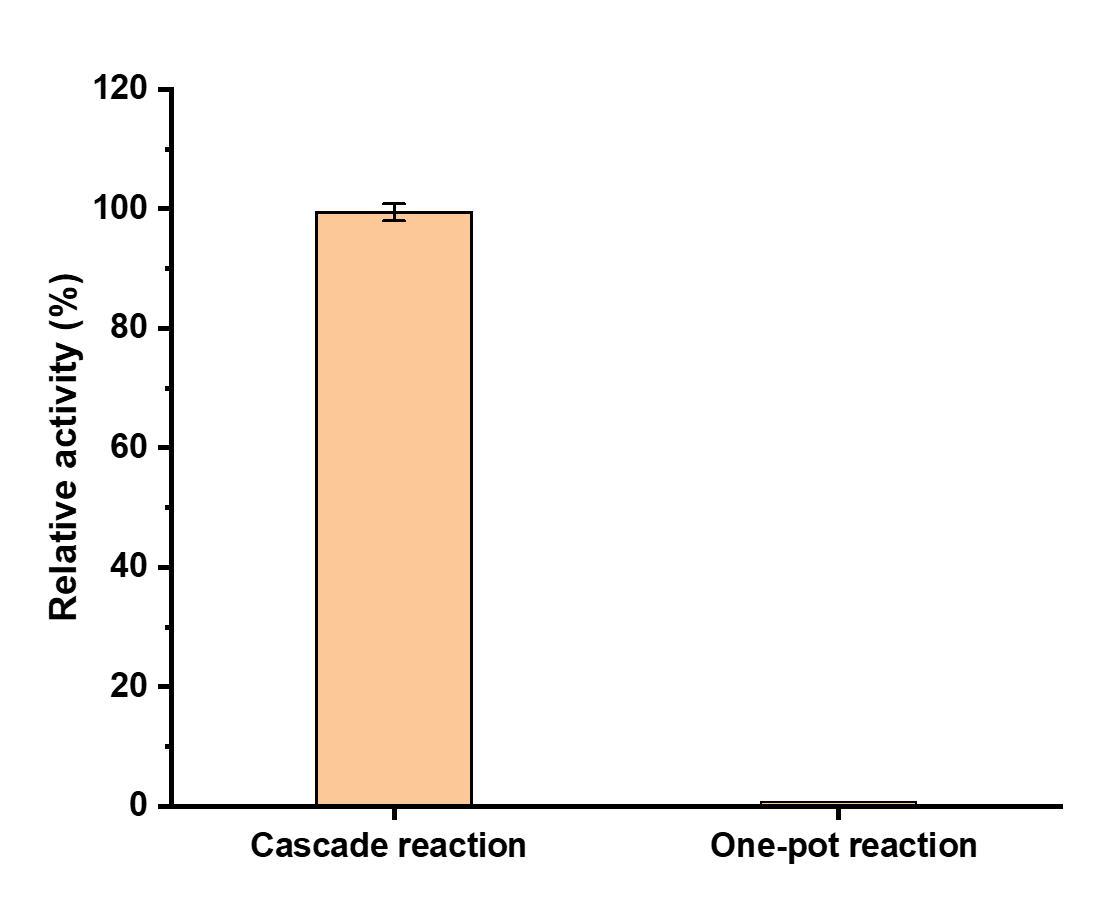


Figure S8. **Comparison of ND*^Mr^* and NST activities under cascade and one-pot reaction conditions.**

In the cascade reaction, NST sequentially catalyzed the products generated by ND*^Mr^*. In contrast, the one-pot reaction involved the simultaneous addition of ND*^Mr^* and NST to the reaction system for heparosan modification. Enzyme activity in the cascade reaction was set as 100% for reference, whereas the one-pot reaction exhibited a lower relative activity.


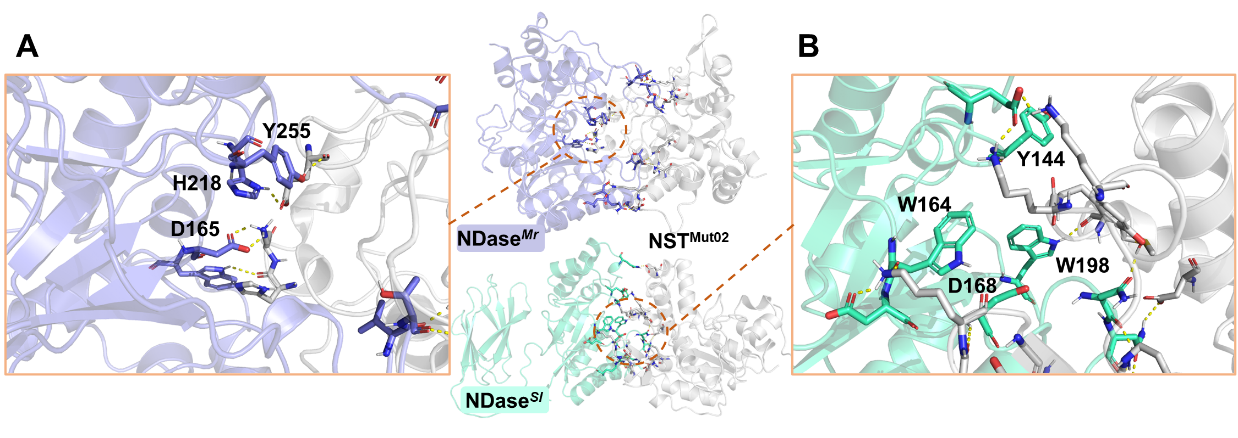


Figure S9. **Structural characterization of the interactions between distinct deacetylases and the *N*-sulfotransferase domain.** Interaction analysis between the NST domain (silver) and the ND*^Mr^* domain (purple) (A) or the ND*^Sl^* domain (cyan) (B). Yellow dashed lines represent the hydrogen bonds.

**
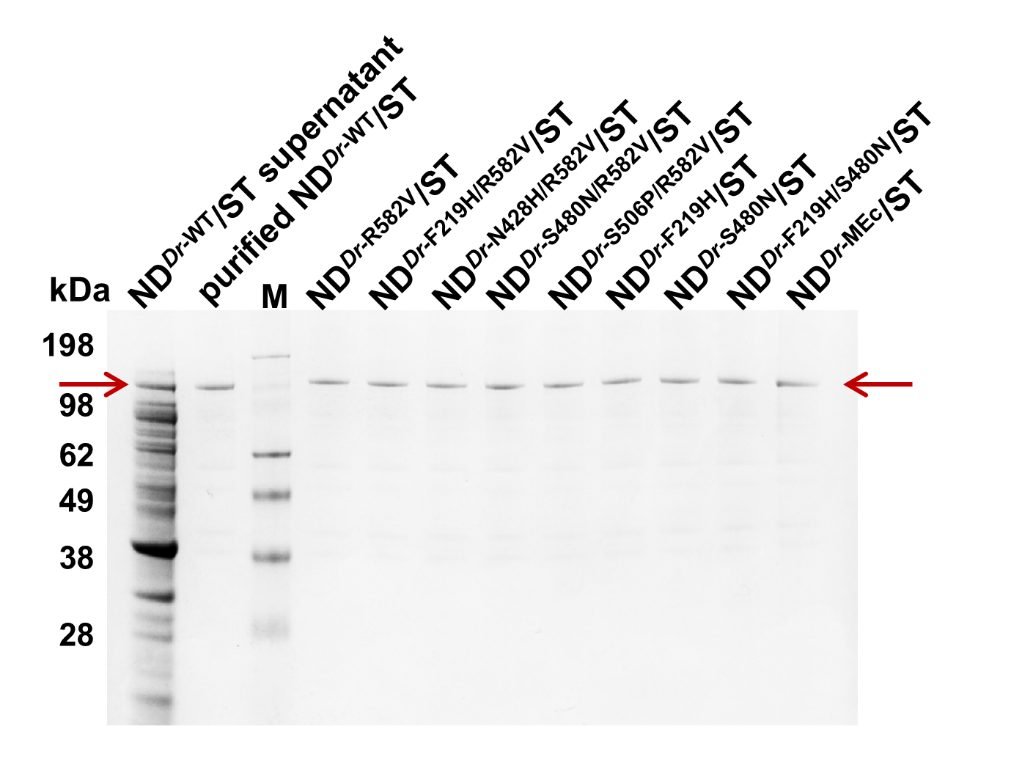
**

Figure S10. SDS-PAGE analysis of ND*^Dr^*/ST WT and its variants.


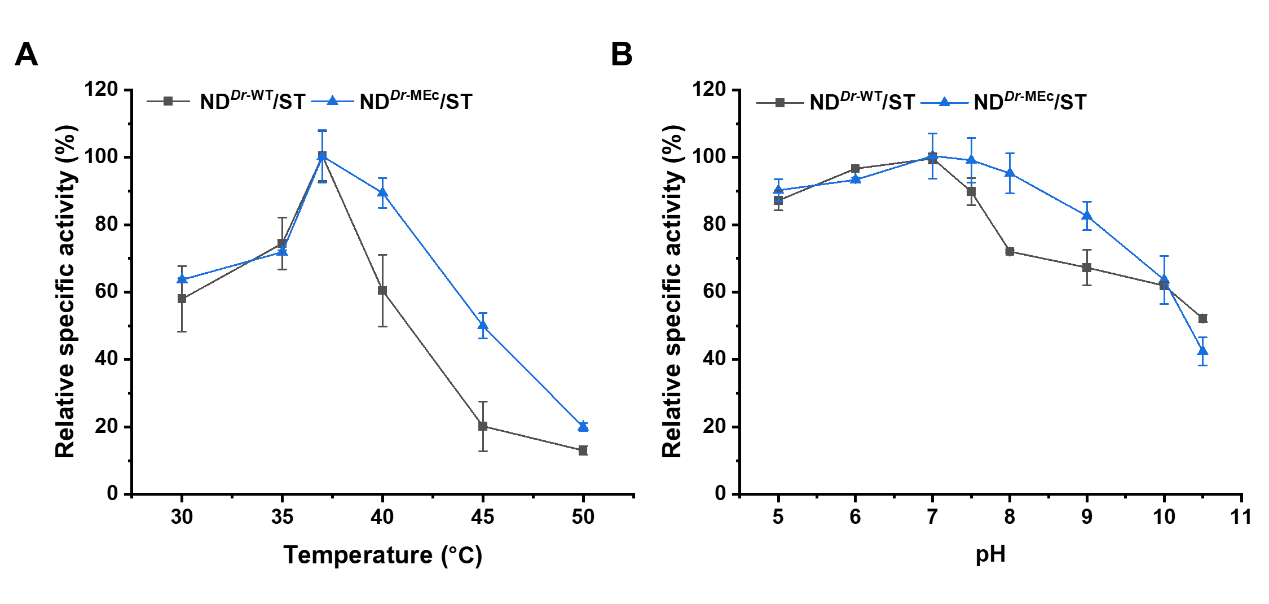


Figure S11. **Enzymatic characterization of ND*^Dr^*^-WT^/ST and ND*^Dr^*^-MEc^/ST.** (A) Optimal reaction temperatures and (B) optimal pH profiles.

Relative specific activities were normalized to the maximum specific activity of each enzyme (set as 100%).

**
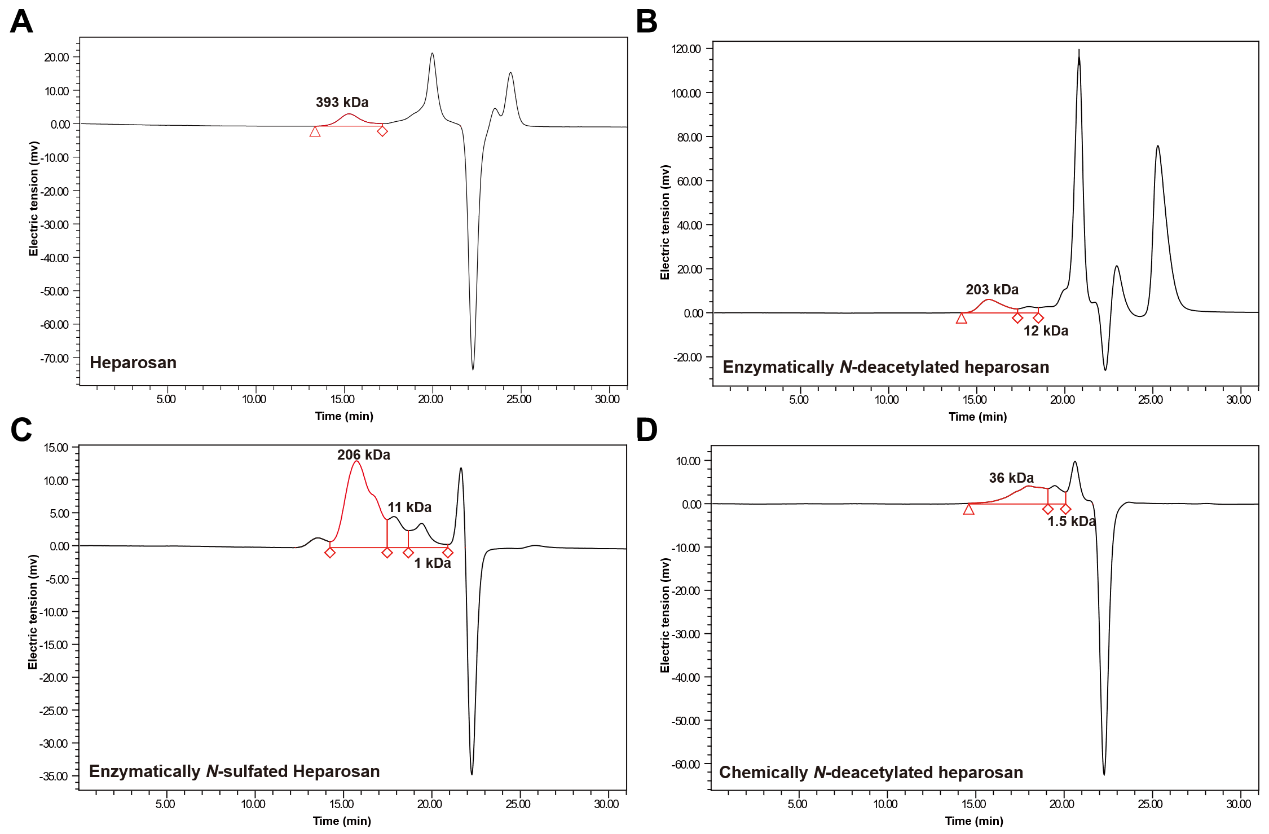
**

Figure S12. **Apparent molecular weight distributions of heparosan and its modified products analyzed by GPC-HPLC.** GPC chromatogram of heparosan (A), enzymatically *N*-deacetylated heparosan generated by ND*^Dr^*^-MEc^/ST in the absence of PAPS (B), enzymatically *N*-sulfated heparosan produced by ND*^Dr^*^-MEc^/ST (C), and chemically *N*-deacetylated heparosan treated with NaOH (D).

Enzymatic reactions yielding *N*-deacetylated heparosan and *N*-sulfated heparosan reactions were terminated by heating at 100 °C for 10 min, followed by dialysis (10 kDa MWCO). Chemically *N*-deacetylated heparosan was prepared by NaOH treatment at 60 °C for 3 h,^[1]^ followed by dialysis (3.5 kDa MWCO), All samples were then followed by lyophilization and dissolution in H_2_O for GPC-HPLC analysis.

**
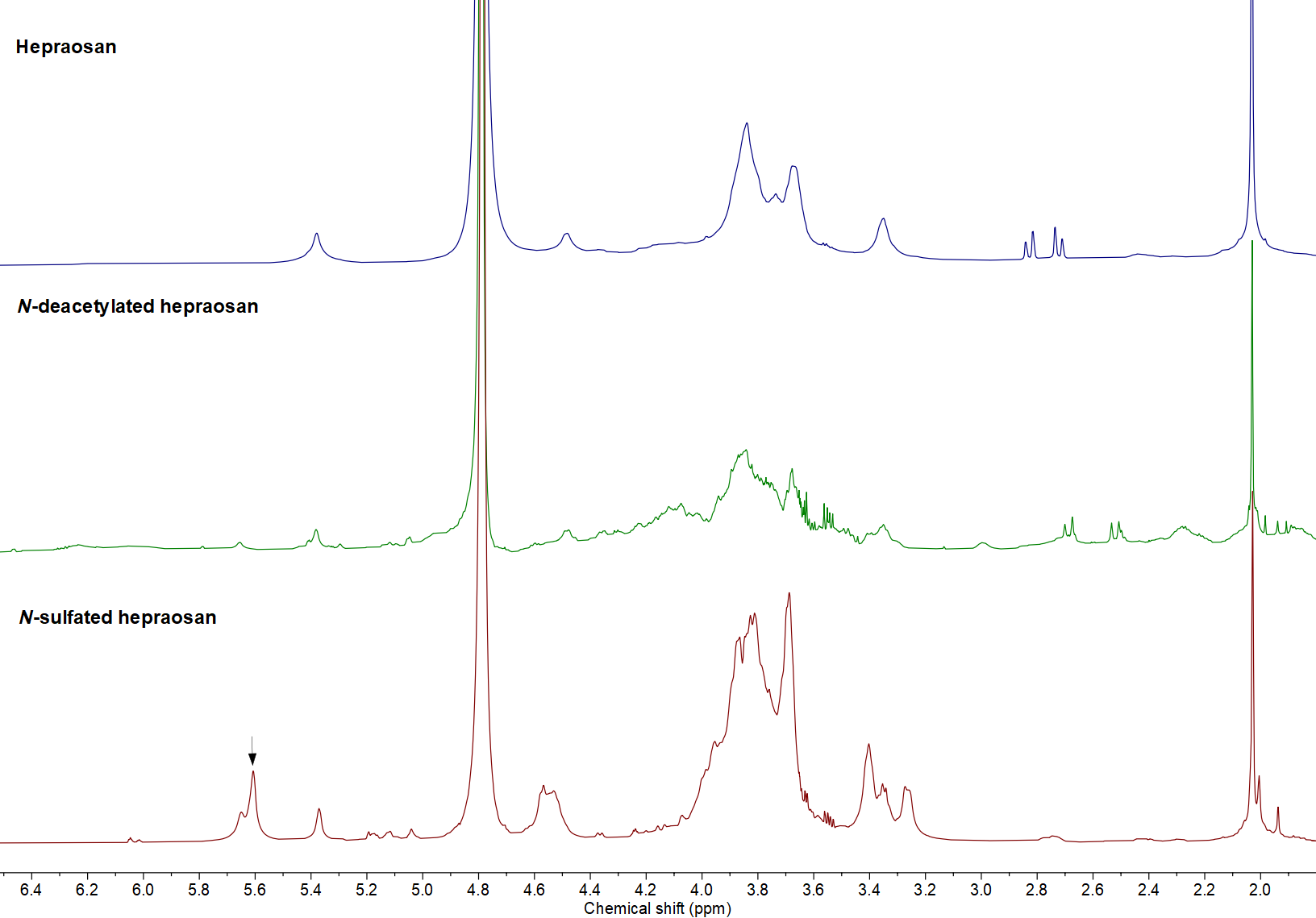
**

Figure S13. **^1^H NMR analysis of heparosan and its modified products.**

**
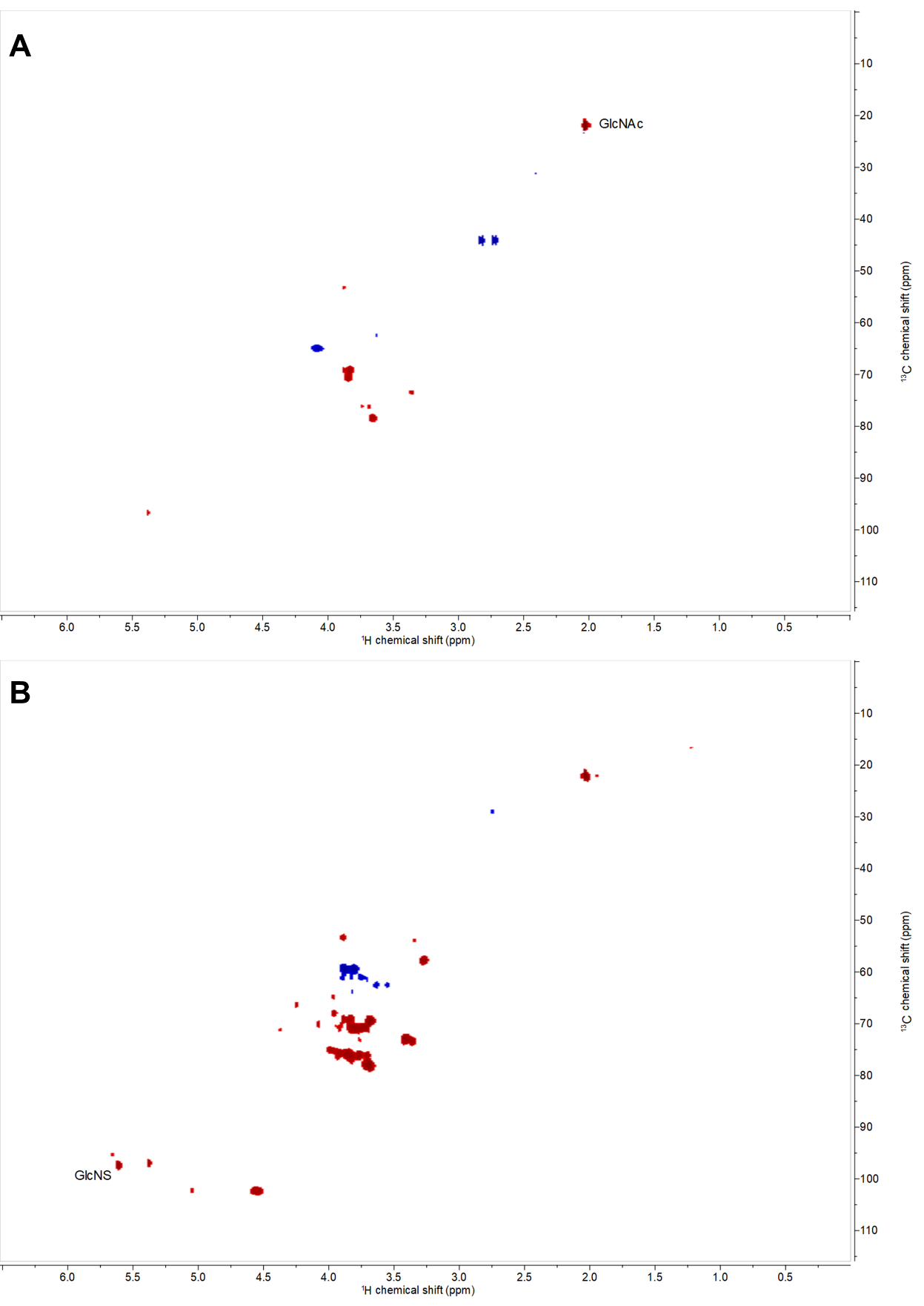
**

Figure S14. **^1^H-^13^C-heteronuclear single quantum coherence analysis of heparosan (A) and *N*-sulfated heparosan (B).**

**
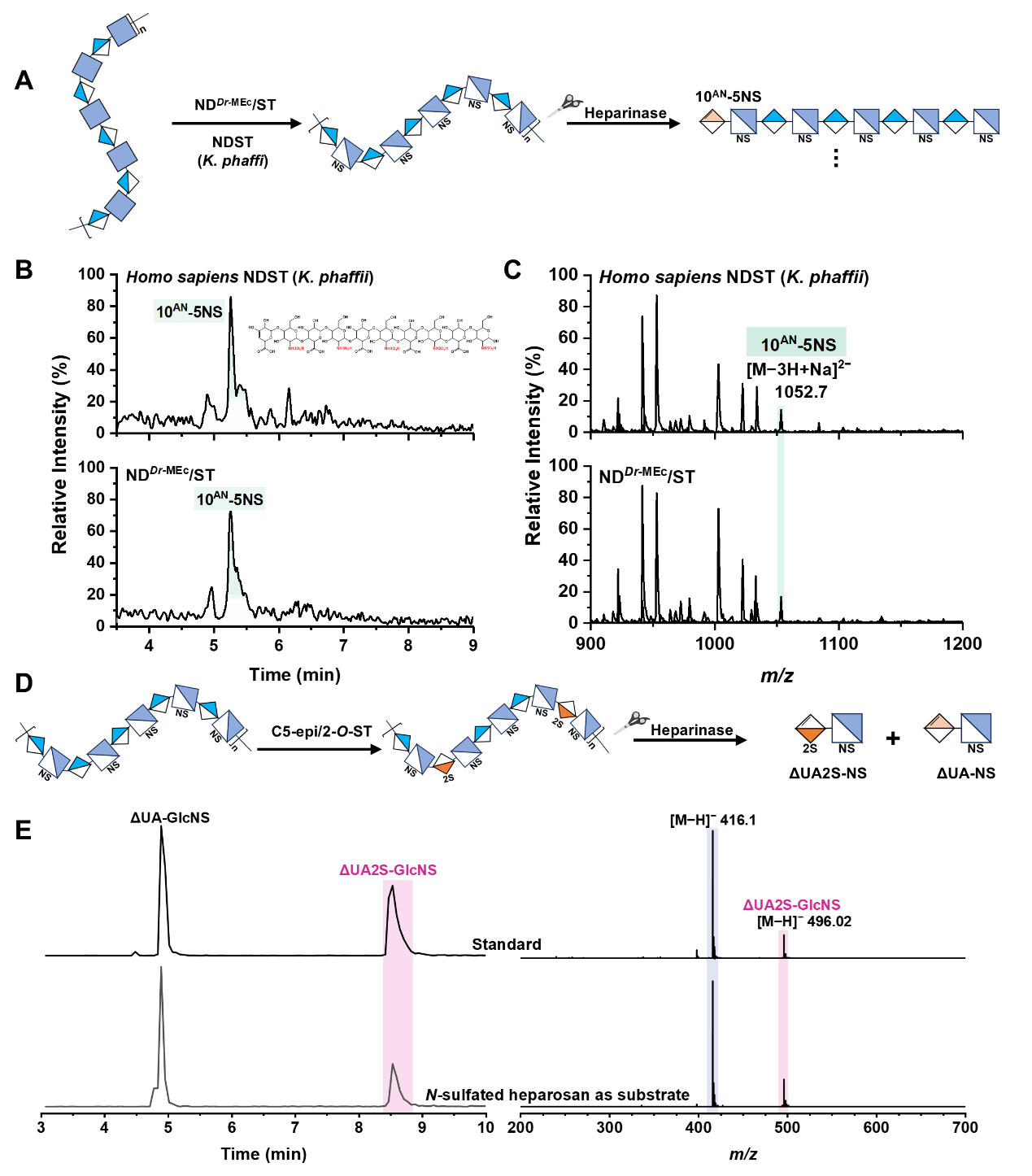
**

Figure S15. **Validation of** ***N*-sulfated heparosan generated by ND*^Dr^*^-MEc^/ST.**

(A) Schematic illustration of the validation of enzymatically synthesized *N*-sulfated heparosan. (B) UPLC chromatograms of heparinase-depolymerized *N*-sulfated heparosan decasaccharides (10^AN^-5NS) generated by ND*^Dr^*^-MEc^/ST and *Homo sapiens* NDST expressed in *Komagataella phaffii* ^[2,3]^. (C) Mass spectrum of 10^AN^-5NS, with a characteristic ion at m/z 1052.7 corresponding to [M–3H+Na]^2−^. (D) Schematic illustration of the downstream enzymatic modification of enzymatically synthesized *N*-sulfated heparosan. (E) UPLC-MS analysis of *N*- and 2-*O*-sulfated heparosan. *N*-sulfated heparosan was further treated with C5-epimerase (C5-epi) and 2-*O*-sulfotransferase (2-*O*-ST)^[4]^ to verify its suitability for downstream enzymatic modification. The characteristic disaccharide ΔUA2S-GlcNS was detected with an [M–H]^−^ ion at m/z 496.02.

**Reference:**

[1] Z. Wang, J. Li, S. Cheong, et al., Response surface optimization of the heparosan *N*-deacetylation in producing bioengineered heparin. *J. Biotechnol.* 2011, *156* (3), 188–196.

[2] Y. Zhang, Y. Wang, Z. Zhou, et al., Synthesis of bioengineered heparin by recombinant yeast Pichia pastoris. *Green Chem.* 2022, 24 (8), 3180–3192.

[3] X. Xi, W. Zhang, L. Hu, et al., Enzymatic construction of a library of even- and odd-numbered heparosan oligosaccharides and their *N*-sulfonated derivatives. *Int. J. Biol. Macromol*. 2024, 264, 130501.

[4] W. Chen, D. Wang, X. Xi, et al., Efficient expression of an engineered heparan sulfate 2-*O*-sulfotransferase with improved catalytic properties. *J. Agric. Food Chem.* 2025, *73* (21), 12856–12866.
